# Effectiveness of Standard Sequential Bilateral Repetitive Transcranial Magnetic Stimulation vs Bilateral Theta Burst Stimulation in Older Adults With Depression: The FOUR-D Randomized Noninferiority Clinical Trial

**DOI:** 10.1001/jamapsychiatry.2022.2862

**Published:** 2022-09-21

**Authors:** Daniel M. Blumberger, Benoit H. Mulsant, Kevin E. Thorpe, Shawn M. McClintock, Gerasimos N. Konstantinou, Hyewon H. Lee, Sean M. Nestor, Yoshihiro Noda, Tarek K. Rajji, Alisson P. Trevizol, Fidel Vila-Rodriguez, Zafiris J. Daskalakis, Jonathan Downar

**Affiliations:** 1 Temerty Centre for Therapeutic Brain Intervention, Campbell Family Research Institute, Centre for Addiction and Mental Health, Toronto, Ontario, Canada; 2 Department of Psychiatry, Temerty Faculty of Medicine, University of Toronto, Toronto, Ontario, Canada; 3 Dalla Lana School of Public Health, University of Toronto, Toronto, Ontario, Canada; 4 Applied Health Research Centre (AHRC), Li Ka Shing Knowledge Institute of St Michael’s Hospital, Toronto, Ontario, Canada; 5 Department of Psychiatry, University of Texas Southwestern Medical Center, Dallas; 6 Harquail Centre for Neuromodulation, Sunnybrook Health Sciences Centre, Toronto, Ontario, Canada; 7 Department of Neuropsychiatry, Faculty of Medicine, Keio University School of Medicine, Tokyo, Japan; 8 Toronto Dementia Research Alliance, University of Toronto, Toronto, Ontario, Canada; 9 Non-Invasive Neurostimulation Therapies (NINET) Laboratory, University of British Columbia Hospital, Vancouver, British Columbia, Canada; 10 Department of Psychiatry, University of British Columbia, Vancouver, British Columbia, Canada; 11 Department of Psychiatry, University of California, San Diego Health

## Abstract

**Importance:**

Treatment-resistant depression (TRD) is common in older adults. Bilateral repetitive transcranial magnetic stimulation (rTMS) of the dorsolateral prefrontal cortex for 48 minutes has demonstrated efficacy in TRD. Theta burst stimulation (TBS), a newer form of rTMS, can also be delivered bilaterally using left intermittent TBS and right continuous TBS for only 4 minutes.

**Objective:**

To establish the effectiveness and tolerability of TBS compared with standard rTMS in older adults with TRD.

**Design, Setting, and Participants:**

In this randomized noninferiority trial with open treatment and blinded assessors, recruitment occurred between December 2016 and March 2020. The trial was conducted at the Centre for Addiction and Mental Health in Toronto, Ontario, Canada and included outpatients 60 years and older with a diagnosis of depression, moderate severity, and nonresponse to 1 or more antidepressant trial of adequate dosage and duration or intolerance of 2 or more trials.

**Interventions:**

Participants were randomized to receive a course of 4 to 6 weeks of either bilateral standard rTMS or TBS.

**Main Outcomes and Measures:**

The primary outcome measure was change in Montgomery-Åsberg Depression Rating Scale; secondary outcome measures included the 17-item Hamilton Rating Scale for Depression, Quick Inventory of Depressive Symptomatology (16-item) (self-report), and dropout rates. A noninferiority margin of 2.75 points was used for the primary outcome. All participants who attained the primary completion point of 4 weeks were analyzed.

**Results:**

A total of 87 participants (mean [SD] age, 67.1 [6.7] years; 47 [54.0%] female) were randomized to standard bilateral rTMS and 85 (mean [SD] age, 66.3 [5.3] years; 45 [52.9%] female) to TBS, of whom 85 (98%) and 79 (93%) were assessed for the primary outcome, respectively, whereas tolerability was assessed in all randomized participants. In the rTMS group, 4 (4.6%) were American Indian, reported other, or preferred not to answer; 5 (5.8%) were Asian; and 78 (89.7%) were White. In the TBS group, 6 (7.1%) were Asian, 2 (2.4%) were Black or reported other, and 77 (90.3%) were White. Mean (SD) Montgomery-Åsberg Depression Rating Scale total scores improved from 25.6 (4.0) to 17.3 (8.9) for rTMS and 25.7 (4.7) to 15.8 (9.1) for TBS (adjusted difference, 1.55; lower 95% CI −0.67), establishing noninferiority for TBS. The all-cause dropout rates were relatively similar between groups (rTMS: 2 of 87 [2.3%]; TBS: 6 of 85 [7.1%]; *P* = .14; χ^2^ = 2.2).

**Conclusions and Relevance:**

In older adults with TRD, bilateral TBS compared with standard bilateral rTMS achieved noninferior reduction in depression symptoms. Both treatments had low and similar dropout rates. Using TBS rather than rTMS could increase access to treatment several-fold for older adults with TRD.

**Trial Registration:**

ClinicalTrials.gov Identifier: NCT02998580

## Key Points

**Question:**

Is bilateral theta burst stimulation noninferior to standard bilateral repetitive transcranial magnetic stimulation (rTMS) in older adults with treatment-resistant depression?

**Findings:**

In this randomized noninferiority trial of 172 participants, improvement on the primary clinical measure (Montgomery-Åsberg Depression Rating Scale) and all secondary clinical outcome measures in patients receiving bilateral theta burst stimulation were robustly noninferior to those receiving standard bilateral rTMS. Additionally, cognitive outcomes and dropout rates were similar between groups.

**Meaning:**

In older adults with treatment-resistant depression, bilateral theta burst stimulation compared with standard bilateral rTMS achieved noninferior reduction in depression symptoms.

## Teaser

This randomized noninferiority trial establishes the effectiveness and tolerability of theta burst stimulation compared with standard repetitive transcranial magnetic stimulation in older adults with treatment-resistant depression.

## Introduction

Depression is the most common and treatable mental disorder in later life, making it a major public health concern,^1^ and 7% of older adults develop major depressive disorder.^2^ Late-life depression is further complicated by comorbid physical illness and is associated with high rates of disability, mortality, and health care utilization.^1^ Importantly, depression is an important treatment target for dementia prevention as it is a modifiable risk factor.^3^

Current pharmacological treatments for late-life depression have well-established but modest efficacy, with rates of nonresponse to first-line antidepressants ranging from 55% to 81%.^1^ Nonresponse to first-line antidepressant treatment in older adult patients contributes to diminished quality of life.^1^ As a result, treatment-resistant depression (TRD) in older adults has been identified as a priority area for research.^4^

Repetitive transcranial magnetic stimulation (rTMS) is a treatment that involves direct stimulation of cortical neurons using focused magnetic field pulses; rTMS of the left side of the dorsolateral prefrontal cortex (DLPFC) at 10 Hz is a well-tolerated, evidence-based treatment for TRD.^5^ However, in older adults, most rTMS studies to date have been limited by suboptimal stimulation parameters, small sample sizes, and insufficient treatment durations.^6^ While optimal parameters for rTMS are still in the process of being established, our group found in 2 separate studies superior remission rates with bilateral stimulation (40%) compared with both left-unilateral (0%) and sham (0%) stimulation in older patients with TRD.^7^

Potential therapeutic advantages of bilateral stimulation can be offset by the need for longer treatment sessions to stimulate both hemispheres sequentially (ie, 30 to 60 minutes). Long sessions reduce treatment capacity; thus, for protocols that involve stimulation of more than 1 target per session, it is particularly desirable to develop techniques for reducing the overall session length. Theta burst stimulation (TBS), which uses patterned bursts of stimulation applied over a fraction of the time than standard treatments,^8^ may be of value in treating older adults with TRD in this regard. TBS may be a more potent physiologic form of stimulation than standard rTMS as it is based on coupling of γ and θ frequency rhythms of the brain.^9^ Preclinical research suggests that TBS can achieve similar or more potent effects on neural plasticity, with durable effects ensuing from 600 pulses of intermittent TBS (iTBS) over 3 minutes or 600 pulses of continuous TBS (cTBS) over 40 seconds.^10^ Previous work has demonstrated that a 3-minute session of 600 pulses of iTBS achieves a similar reduction in depressive symptoms^9^ compared with the standard US Food and Drug Administration (FDA)–cleared 10-Hz rTMS protocol.^11^ Bilateral TBS protocols have also been studied in major depressive disorder, and they yield higher remission rates than sham TBS^12^ while still reducing session duration.

As such, we conducted a randomized noninferiority trial to investigate the relative effectiveness of standard sequential bilateral rTMS (47.5 minutes) vs bilateral TBS (4 minutes) in older adults with TRD. A noninferiority rather than equivalence design was chosen because of the asymmetric time advantage of TBS. We hypothesized that bilateral TBS would be noninferior in reducing depressive symptoms compared with standard bilateral rTMS. Additionally, we compared the effects of TBS vs standard rTMS on cognition, self-reported adverse effects (including pain), and all-cause dropout rates.

## Methods

### Setting and Participants

The trial was conducted at an academic health center (Centre for Addiction and Mental Health, Toronto, Ontario, Canada) after research ethics board approval. The trial protocol is available in Supplement 1. All participants provided written, informed consent to participate. From December 2016 to March 2020, we recruited outpatient adults 60 years and older with major depressive disorder confirmed by the Mini-International Neuropsychiatric Interview,^13^ moderate severity episode based on a Montgomery-Åsberg Depression Rating Scale (MADRS)^14^ score of 18 or higher, nonresponse to 1 or more antidepressant trial of adequate dosage and duration using the Antidepressant Treatment History Form^15^ or intolerance of 2 or more antidepressants, no increase or initiation of psychotropic medication 4 weeks prior to screening, and normal prestudy blood work results. Exclusion criteria were substance misuse or dependence within the last 3 months, unstable physical illness, active suicidal intent, current psychotic symptoms, bipolar disorder, other psychiatric disorder causing greater impairment than major depressive disorder, dementia, Short Blessed Test^16^ total score of more than 10, electroconvulsive therapy or rTMS during the current episode, seizure disorder or lesion-related seizure, intracranial implant or metal in the cranium, implanted electronic device, and anticonvulsant use or benzodiazepine more than or equal to lorazepam 2 mg/d equivalents. During the treatment course, preexisting pro re nata medications were allowed; pro re nata benzodiazepine use was discouraged and had to be within the allowable limits. Race and ethnicity were collected by self-report.

### Randomization and Blinding

Outpatients were randomized by personnel external to the clinical team using a computer-based algorithm and allocated in a 1:1 ratio to the standard bilateral protocol or the bilateral TBS protocol. Randomly permuted blocks, with stratification by level of treatment resistance (0 or 1 vs >1 adequate trial). Nontransparent, sealed envelopes with a randomization identification on the outside and treatment assignment on the inside were used for allocation. The randomization identification was assigned, and treatment allocation was accessed immediately prior to the first session by the treating technician. Both technicians and participants (because of the different duration of sessions) were aware of the treatment condition. Participants were instructed not to discuss their treatment with others in the clinic. Outcome raters were blinded to treatment assignment and located on another floor.

### Standard rTMS and TBS Procedures

Following baseline magnetic resonance imaging (MRI), participants underwent neuronavigation using the Visor 2 system (Advanced Neuro Therapeutics) to position the transcranial magnetic stimulation (TMS) coil at a minimum distance from a left-sided DLPFC target coordinate defined based on anticorrelation to the subgenual cingulate cortex and its right-hemisphere counterpart (MNI-152 stereotaxic coordinate [x ± 38 y + 44 z + 26]).^17^ Participants who could not tolerate the MRI (because of claustrophobia) were allowed to undergo treatment using an approximated method.^18^

Treatment was delivered with a MagPro X100 stimulator equipped with the B70 fluid-cooled coil (MagVenture). Standard sequential bilateral rTMS consisted of 1-Hz stimulation (120% resting motor threshold [RMT], 600 pulses over 10 minutes) to the right DLPFC, followed by standard FDA-cleared 10-Hz stimulation (120% RMT, 3000 pulses: 4 seconds on, 26 seconds off over 37.5 minutes) to the left DLPFC. Sequential bilateral TBS was delivered at the same stimulation sites and intensity (120% RMT) with right-sided cTBS (triplet burst pulses at 50 Hz, repeated at 5 Hz for 600 pulses over 40 seconds) followed by FDA-cleared left-sided iTBS (triplet burst pulses at 50 Hz, repeated at 5 Hz, 2 seconds on, 8 seconds off, for 600 pulses over 3 minutes 9 seconds). An adaptive titration to 120% RMT was used within the first 4 treatments to aid with tolerability.

As done in clinical practice, each participant’s RMT was determined using visual observation.^5^ Participants received 20 initial daily sessions over 4 weeks and were offered an additional 10 daily sessions over 2 additional weeks if they did not achieve remission after 20 sessions. Participants who missed scheduled treatment days due to intercurrent illness or scheduling conflicts received the entire 20 to 30 treatments over a longer time period. Participants missing more than 3 consecutive treatments were withdrawn.

### Clinical Measures

Clinical outcome measures were assessed at baseline, after every 5 sessions, and then 1, 4, and 12 weeks after treatment. MADRS score was the primary outcome measure from baseline to end of treatment. Secondary depression symptom severity outcome measures included the 17-item Hamilton Rating Scale for Depression (HRSD-17)^19^ and the Quick Inventory of Depressive Symptomatology (16-item) (self-report) (QIDS-SR-16).^20^ Response rates (defined as ≥50% score improvement from baseline) and remission rates (defined as MADRS score ≤10, HRDS-17 score ≤ 7, and QIDS-SR-16 score ≤ 5) were secondary outcome measures. To assess other potential characteristics associated with response to rTMS, we obtained the Brief Symptom Inventory–anxiety^21^ and the Cumulative Illness Rating Scale for Geriatrics^22^ scores.

### Safety and Tolerability Measures

Because cognition is typically impaired in late-life depression^23^ and an important predictor of antidepressant outcome,^24^ a cognitive battery was administered (baseline, end of treatment, and 12 weeks posttreatment). Executive function was assessed with the Delis-Kaplan Executive Function System Color-Word Interference test^25^ and the National Institutes of Health Toolbox Flanker Inhibitory Control and Attention Test.^26^ Memory was assessed with the California Verbal Learning Test–second edition.^27^ Global cognition was assessed with 3 different versions of the Montreal Cognitive Assessment.^28^

At each session, self-reported adverse effects were queried, and participants self-rated pain intensity on a verbal analogue scale (0 = no pain, 10 = intolerable pain). We recorded the number of sessions required to reach 120% RMT by session end and number of sessions required to start the session at this target intensity. Serious adverse events (SAEs) and reasons for treatment discontinuation were recorded.

### Statistical Analysis

According to the National Institute of Clinical Health and Excellence, a clinically meaningful difference between drug and placebo is defined by 3 points or more on a depression rating scale.^29^ For the primary analysis, based on our previous rTMS trial in older adults with TRD,^7^ we used a δ of 2.75 and a standard deviation of change of 7.8 and estimated a sample size of 200 participants was required to determine if bilateral TBS was noninferior to bilateral rTMS with 80% power and a 1-sided alpha of 0.05. As intention-to-treat analyses can bias in favor of noninferiority, the primary analysis included participants who had completed the majority of 4 weeks of treatment.^30^ Two sensitivity analyses were conducted: one excluded 7 participants who were not able to receive an MRI and the other used the intention-to-treat sample.

To assess the primary outcome analysis, baseline-adjusted change was estimated from an analysis of covariance model using final MADRS score as the outcome and baseline score as the adjustment covariate with a δ = 2.75. The null hypothesis was baseline-adjusted mean final MADRS score for standard bilateral rTMS would be at least 2.75 points better than bilateral TBS, and the alternative (noninferiority) hypothesis that baseline-adjusted mean final MADRS score for bilateral rTMS would be less than 2.75 points better than bilateral TBS. The same noninferiority δ = 2.75 was used for HRDS-17 and QIDS-SR-16 scores. Based on raw mean difference between active and sham rTMS for response (21% difference) and remission (14% difference) from a previous meta-analysis,^31^ a noninferiority δ = 15% was used to compare response rates and a noninferiority δ = 10% was used to compare remission rates. Linear mixed-effect models were used to evaluate differences on the cognitive measures.

For tolerability comparisons, participants’ mean pain score across all treatment sessions were calculated for each group and compared. The prevalence of reported adverse effects and the proportion of participants reporting adverse effects were compared. Finally, rates of all-cause dropout from all causes were compared using Fisher exact test. The analysis was conducted with R version 4.1.2 (R Foundation) and the lme 4 package v1.1-29.

## Results

**Figure 1.**
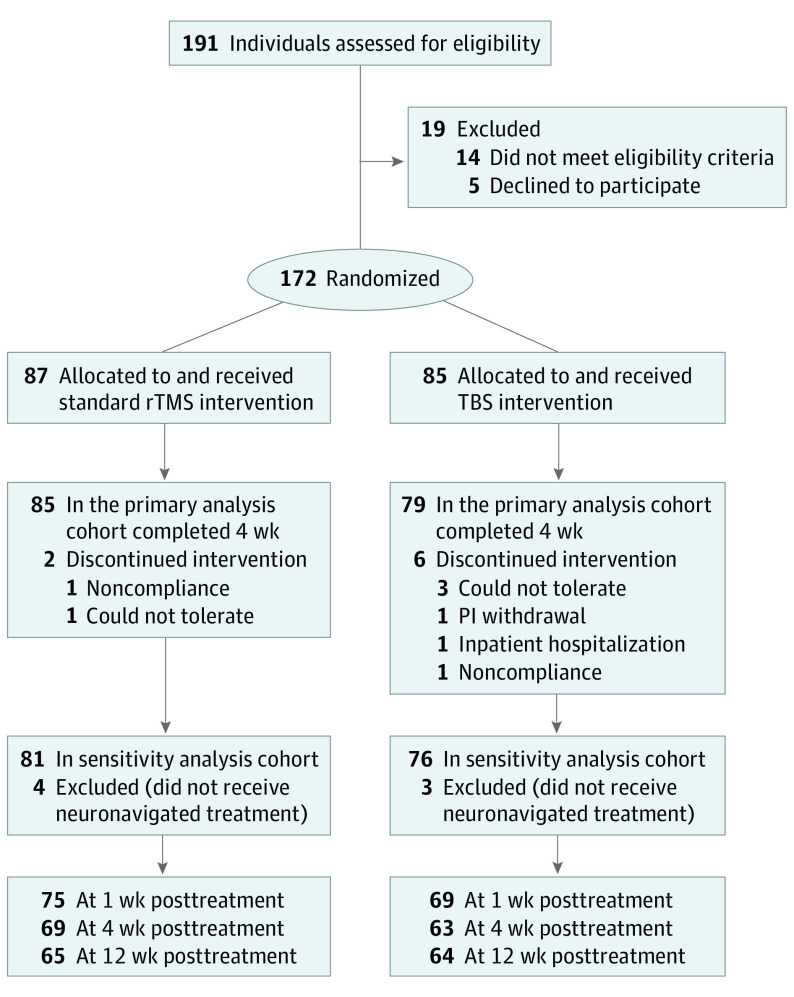
CONSORT Flow Diagram PI indicates principal investigator; rTMS, repetitive transcranial magnetic stimulation; TBS, theta burst stimulation.

Table 1 summarizes the characteristics of the intention-to-treat sample of 172 participants randomized to receive treatment by the early stages of the COVID-19 pandemic in March 2020; new recruitment was halted when it became infeasible due to persistent public health restrictions in the province of Ontario, Canada. A total of 164 participants completed the greater part of 4 weeks of treatment and were included in the primary analysis (Figure 1). A total of 87 participants (mean [SD] age, 67.1 [6.7] years; 47 [54.0%] female) were randomized to standard bilateral rTMS and 85 (mean [SD] age, 66.3 [5.3] years; 45 [52.9%] female) to TBS. In the rTMS group, 4 (4.6%) were American Indian, reported other, or preferred not to answer; 5 (5.8%) were Asian; and 78 (89.7%) were White. In the TBS group, 6 (7.1%) were Asian, 2 (2.4%) were Black or reported other, and 77 (90.3%) were White.

**Table 1.** Baseline Demographic and Clinical Characteristics

Characteristic	Mean (SD)	
	rTMS (n = 87)	TBS (n = 85)
Age, y	67.1 (6.7)	66.3 (5.3)
Female, No. (%)	47 (54.0)	45 (52.9)
Male, No. (%)	40 (46)	40 (47.1)
Years of education	15.38 (2.3)	15.16 (3.0)
MADRS score	25.7 (4.5)	26 (4.9)
HRSD-17 score	18.3 (4.0)	18.7 (4.8)
QIDS score	15.6 (4.5)	16.1 (4.5)
BSI-A score	11.0 (5.6)	10.0 (5.3)
Age at onset, y	30.2 (19.3)	31.6 (18.4)
Episode duration, mo	50.2 (82.3)	71.5 (109.8)
History of ECT, No. (%)	14 (16.1)	8 (9.4)
History of rTMS, No. (%)	15 (17.2)	12 (14.1)
Psychotherapy during the episode, No. (%)	30 (34.5)	28 (32.9)
Pharmacotherapy during treatment, No. (%)		
No antidepressant medication	11 (12.6)	13 (15.3)
Hypnotic Z drugs	20 (23.0)	14 (16.5)
Benzodiazepine use	34 (39.1)	42 (49.4)
Antidepressant alone	36 (41.4)	30 (35.3)
Antidepressant combination	20 (23.0)	13 (15.3)
Antipsychotic augmentation	21 (32.1)	19 (22.4)
Lithium augmentation	2 (2.3)	6 (7.1)
Antipsychotic alone	1 (1.1)	2 (2.4)
Stimulant	7 (8)	7 (8.2)
Cognitive measures		
Baseline flanker task score	89.1 (10.3)	89.5 (9.0)
Baseline DKEFS score		
Color naming task scaled score	8.6 (3.6)	9.5 (3.0)
Color word task scaled score	10.3 (3.0)	10.6 (2.4)
Inhibition scaled score	10.1 (2.9)	10.4 (2.9)
Inhibition/switching scaled score	10.7 (2.9)	11.1 (2.5)
Baseline CVLT-II score		
List A Trials 1-5 T-Score	47.3 (10.7)	47.8 (10.9)
List A Short-Delay Free Recall Z score	−0.17 (1.12)	−0.13 (1.17)
List A Long-Delay Free Recall Z score	−0.28 (1.11)	−0.25 (1.19)
Baseline MOCA score	24.8 (3.7)	25.1 (3.3)
Previous treatment history, No. (%)		
Unable to tolerate 2 trials	6 (6.9)	6 (7.1)
1 Failed trial	26 (29.9)	25 (29.4)
2 Failed trials	23 (26.4)	27 (31.8)
3 Failed trials	17 (19.5)	15 (17.6)
≥4 Failed trials	15 (17.2)	12 (14.1)

Abbreviations: BSI-A, Brief Symptom Inventory–Anxiety; CVLT-II, California Verbal Learning Test (Second Edition); DKEFS, Delis-Kaplan Executive Function System; ECT, electroconvulsive therapy; HRSD-17, 17-item Hamilton Depression Rating Scale; MADRS, Montgomery-Åsberg Depression Rating Scale; MOCA, Montreal Cognitive Assessment; QIDS, Quick Inventory of Depressive Symptomatology; rTMS, repetitive transcranial magnetic stimulation; TBS, theta burst stimulation.

### Efficacy Outcomes

#### Primary Analysis

**Figure 2.**
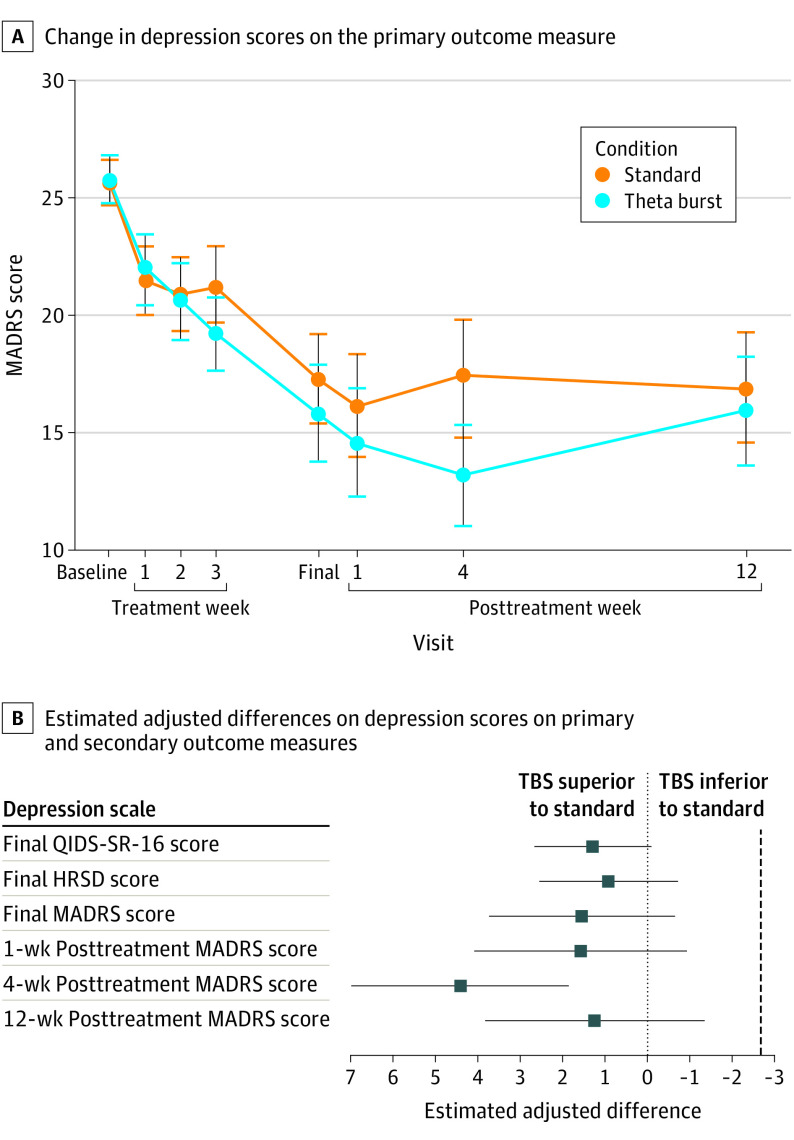
Relative Change and Differences in Depression Scores A, Change in Montgomery-Åsberg Depression Rating Scale (MADRS) scores over time in the standard bilateral repetitive transcranial magnetic stimulation (n = 85) and bilateral theta burst stimulation (TBS) (n = 79) groups. Points represent mean scores and bars represent 90% CI. B, Estimated adjusted difference shown with 2-sided lower and upper 90% CIs, though the test of noninferiority was conducted using a 1-side lower 95% CI. The dotted line represents the noninferiority *δ* = −2.75 points. See Table 2 for estimated adjusted differences and tests of noninferiority. HRSD indicates 17-item Hamilton Rating Scale for Depression; QIDS-SR-16, Quick Inventory of Depressive Symptomatology (16-item) (self-report).

Final MADRS score change showed an estimated adjusted difference of 1.55 points favoring TBS, with a lower 95% CI of −0.67, lower than the a priori noninferiority *δ = *2.75 points. Additional analyses demonstrated noninferiority at all follow-up time points (eTable 1 in Supplement 2) and in the sensitivity analysis sample (n = 157) (estimated adjusted difference of 1.22 points favoring TBS, with a lower 95% CI of −1.06 points). A comparison of the change in MADRS scores over time for the 2 groups is depicted in Figure 2A and the relative difference in change compared to the noninferiority margin in Figure 2B.

#### Secondary Outcome Analyses

On all secondary outcome measures, TBS also demonstrated noninferiority to standard bilateral rTMS (Table 2) and at all follow-up time points (eTable 1 in Supplement 2). The MADRS response rate for TBS was 44.3% (35 of 79) compared with 32.9% (28 of 85) for standard bilateral rTMS with an estimated adjusted difference of 11.4% favoring TBS, with a lower 95% CI of −1.1%, smaller than the noninferiority *δ < *15%. Similarly, the MADRS remission rate for TBS was 35.4% (28 of 79) compared with 32.9% (28 of 85) for standard bilateral rTMS with an estimated adjusted difference of 2.5% favoring TBS, with a lower 95% CI of −9.7%, smaller than the noninferiority *δ < *10%.

**Table 2.** Response, Remission, and Change in Depression Severity Scores^a^

Outcome measure	Mean (SD)		Estimated adjusted difference, % (90% CI)^b^	*P* value^b^
	rTMS (n = 85)	TBS (n = 79)		
**MADRS**				
Baseline score	25.6 (4.5)	25.7 (4.7)	NA	NA
Final	17.3 (8.9)	15.8 (9.1)	1.547 (−0.66 to 3.75)	<.001
Response rate, No. (%)	28 (32.9)	35 (44.3)	11.40 (−1.10 to 23.80)	<.001
Remission rate, No. (%)	28 (32.9)	28 (35.4)	2.50 (−9.70 to 14.70)	.046
**HRSD-17**				
Baseline	18.4 (4.1)	18.5 (4.7)	NA	NA
Final	12.3 (6.8)	11.4 (6.8)	0.917 (−0.72 to 2.56)	<.001
Response rate, No. (%)	24 (29.6)	31 (41.9)	12.30 (−0.30 to 24.90)	<.001
Remission rate, No. (%)	22 (27.2)	25 (33.8)	6.60 (−5.50 to 18.80)	.01
**QIDS-16**				
Baseline	15.7 (4.4)	15.9 (4.4)	NA	NA
Final	10.8 (6.0)	9.7 (6.1)	1.268 (−0.1 to 2.67)	<.001
Response rate, No. (%)	30 (35.7)	35 (44.3)	8.60 (−4.00 to 21.20)	.001
Remission rate, No. (%)	18 (21.4)	25 (31.6)	10.20 (−1.10 to 21.50)	.002
BSI-A baseline	11.1 (5.6)	9.8 (5.2)	NA	NA
BSI-A final	6.6 (5.8)	5.7 (5.2)	0.341 (−0.99 to 1.67)	<.001

Abbreviations: BSI-A, Brief Symptom Inventory–anxiety; HRSD-17, 17-item Hamilton Rating Scale for Depression; MADRS, Montgomery-Åsberg Depression Rating Scale; NA, not applicable; QIDS-16, 16-item Quick Inventory of Depressive Symptomatology; rTMS, repetitive transcranial magnetic stimulation; TBS, theta burst stimulation.

^a^ For estimated adjusted difference values, positive values indicate greater change in the bilateral TBS group, while negative values indicate greater change in the standard bilateral rTMS group.

^b^ Corresponds to the lower 95% CI of the 1-sided test for noninferiority. *P* values indicate the significance of rejecting the null hypothesis based on the change in symptoms in the 2 groups and on a noninferiority δ of 15% for the proportion of responders and 10% for the proportion of remitters.

#### Cognitive Safety

There were no substantial changes on any of the cognitive measures and no significant differences between standard rTMS and TBS on any of the cognitive measures (eTable 3 in Supplement 2).

#### Tolerability Outcomes

The number of reported adverse events for headache, nausea, dizziness, or other adverse events were relatively similar between standard rTMS and TBS (Table 3). Pain scores were significantly lower in the rTMS than in the TBS group (mean [SD]: right side, 1.66 [1.79] for standard rTMS and 3.59 [2.45] for TBS; left side, 2.80 [1.99] for standard rTMS and 4.01 [2.55] for TBS). The number of days to reach target intensity on the right side was also higher in the TBS group (mean [SD]: TBS, 4.6 [4.1] days vs standard rTMS, 2.9 [3.5] days).

**Table 3.** Tolerability

Variable	Participants who experienced each adverse reaction, No. (%)^a^	
	rTMS (n = 87)	TBS (n = 85)
Headache	49 (56.3)	46 (54.1)
Nausea	6 (6.9)	7 (8.2)
Dizziness	17 (19.5)	18 (21.2)
Unrelated medical problem	37 (42.5)	34 (40)
Fatigue	5 (5.7)	5 (5.9)
Insomnia	2 (2.3)	1 (1.2)
Anxiety/agitation	7 (8.0)	8 (9.4)
Back/neck pain	6 (6.9)	9 (10.6)
Unrelated accidents	0 (0)	0 (0)
Vomiting	0 (0)	0 (0)
Tinnitus	2 (2.3)	0 (0)
Migraine aura	4 (4.6)	3 (3.5)
Abnormal sensations	2 (2.3)	2 (2.4)

Abbreviations: rTMS, repetitive transcranial magnetic stimulation; TBS, theta burst stimulation.

^a^ *P* > .05 on Fisher exact tests for each pair of proportions. Participants were queried at each treatment about adverse reactions.

#### Early Discontinuation and SAEs

Two of 87 participants undergoing rTMS (2.3%) and 6 of 85 participants undergoing TBS (7.1%) discontinued treatment prior to the primary end point (Figure 1). During the intervention period, SAEs occurred in 0 of 87 participants undergoing rTMS and 1 of 85 participant undergoing TBS (1.2%) (psychiatric admission for depression); during the postintervention follow-up, SAEs occurred in 2 of 87 participants undergoing rTMS (2.3%) (2 hospitalizations for surgery) and 1 of 85 participant undergoing TBS (1.2%) (psychiatric admission for depression).

## Discussion

To our knowledge, we report on the largest randomized clinical trial of nonconvulsive neuromodulation in older adults with TRD. For these patients, our findings provide strong evidence that TBS is noninferior to standard rTMS in reducing depressive symptoms. Noninferiority was demonstrated on all clinician-rated and self-report psychiatric symptom measures at all assessment points, including 12 weeks after treatment. Both rTMS and TBS demonstrated cognitive safety, with improvement consistent with possible practice effects. There were no differences in self-reported adverse effects or in SAEs. Pain ratings were significantly higher with TBS, particularly with right-sided cTBS where stimulation was delivered in a 40-second continuous train. However, this did not lead to a higher dropout rate. Taken together, these findings suggest that 4-minute sequential bilateral TBS is comparable with standard 47.5-minute bilateral rTMS in older adults with TRD. Importantly, both bilateral treatment protocols incorporated an FDA-cleared left-sided treatment (10-Hz rTMS or iTBS) preceded by right-sided priming (with 1-Hz rTMS or cTBS).

The remission rate of 35.4% for the bilateral TBS group is clinically important in this sample of older adults with TRD and a mean of 2 antidepressant trials (some with more than 4 trials) and some with substantial cognitive impairment. This remission rate is comparable with those seen in our previous bilateral rTMS trial (33%)^7^ and deep rTMS trial (40%)^32^ and comparable with the remission rate seen with aripiprazole augmentation in older adults with TRD.^33^ In addition, this remission rate is consistent with the remission rate of iTBS (32%) in the THREE-D trial in younger adults with TRD.^9^ Standard bilateral rTMS has been shown to be efficacious in multiple clinical trials and was one of the most effective rTMS protocols in 2 recent network meta-analyses.^34,35^ Both the degree of improvement and the remission rate in our bilateral standard rTMS arm are consistent with results of previous randomized clinical trials and provides assay sensitivity. The present study used bilateral stimulation, rather than the unilateral stimulation in the THREE-D trial, based on data suggesting better response to bilateral stimulation compared with sham in older adults^7^ and data demonstrating efficacy of bilateral cTBS/iTBS compared with sham in adults.^12,36^ However, recent clinical registry data indicate no added benefit for bilateral over unilateral stimulation in a lifespan sample.^37^ Thus, although the addition of 40 seconds of right-sided cTBS to 3 minutes of left-sided iTBS is a minimal increase in treatment time, it may also prove to yield minimal increase in clinical benefit over left-sided iTBS in the community setting. Bio-creep has been raised as a concern in serial noninferiority trials^38^; the fact that the TBS arm performed better than the standard measure on all measures and outcomes argues against this phenomenon occurring here. Indeed, the TBS arm demonstrated an ongoing improvement up to 4 weeks posttreatment that was superior to standard rTMS. This finding of sustained and progressive improvement is consistent with the possibility that TBS induces greater brain plasticity than standard rTMS protocols.^10^

Both treatments were well tolerated and had high retention during active treatment (95% retention). As in the THREE-D trial, TBS was associated with higher pain ratings, more pronounced for cTBS. However, this did not translate into a higher dropout rate possibly because of the much shorter duration of stimulation with TBS. Importantly, both treatments showed cognitive safety, with almost all measures demonstrating relatively improved performance after treatment that was sustained in those assessed 12 weeks after treatment.

The strong noninferiority findings replicate the THREE-D findings in a vulnerable patient population with currently limited antidepressant treatment options. Previous work has suggested that older adults require higher stimulation intensities^39^ to ensure sufficient cortical stimulation. Therefore, we ensured that both groups received stimulation at 120% RMT. While this intensity is consistent with current recommendations for standard rTMS,^5^ there is no current consensus on the optimal intensity for TBS and other studies have used lower intensities.^12^ A recent trial suggested similar outcomes for TBS at 80% vs 120% RMT.^40^ However, the study enrolled mixed-age adults rather than only older adults, as we did. Thus, it remains unclear whether TBS at lower intensities is suitable in older adults who may have a greater degree of prefrontal atrophy.

### Limitations

Several limitations should be considered. The study was designed to measure the effectiveness of standard sequential bilateral rTMS and bilateral TBS in a real-world sample of older adults with TRD. As such, there was no sham group and participants were aware of their treatment allocation. The decision to compare TBS with standard bilateral TMS rather than sham was a recognition of network meta-analytic findings supporting bilateral standard TMS over sham treatment^34,35^ as well as the widespread use of bilateral standard TMS in the community setting.^37^ However, a sham arm would have allowed for an assessment of the contribution of nonspecific factors. The longer duration of standard treatment involved substantially longer therapeutic contact with technicians, which could have added nonspecific bias in favor of standard rTMS. Despite this relative disadvantage experienced by those in the TBS group, the overall outcomes demonstrated noninferiority and strengthen the overall findings. The possibility that therapeutically important but nonspecific aspects of TMS treatment (ie, routine establishment, behavioral activation, social interaction) might accrue even with brief appointments is encouraging and bears future enquiry. While the trial is one of the largest neurostimulation trials in older adults with TRD, it was nevertheless conducted at a single site. As a result of the COVID-19 pandemic, trial recruitment was halted for several months. When restarting clinical trials was permitted, prolonged and stringent public health measures in Ontario made resuming the trial infeasible. Furthermore, marked changes in the psychosocial and health care environment associated with the pandemic would have introduced confounding factors between pre– and post–COVID-19 cohorts. Despite the truncated recruitment period and the smaller sample than planned, the findings strongly support the noninferiority of TBS and it is highly unlikely that these findings would change with an additional 20 or 30 participants. Similarly, while we prespecified a 1-sided 95% CI for the analysis and more stringent recommendations suggest a 1-sided 97.5% CI, noninferiority findings remained on all measures with this more stringent criterion (eTable 2 in Supplement 2). The use of MRI neuronavigation is a potential limitation to generalizability, although findings did not change with participants who received approximated treatment with the approximated scalp-based method.^18^ Given that the clinical effectiveness seen in the present study lies in a range similar to that reported in large community-based samples,^41^ future studies may wish to reconsider whether MRI-guided neuronavigation adds additional value over scalp-based heuristics^18^ when the stimulation target is defined from population-based coordinates rather than individualized functional neuroimaging data.^42^ While the left-sided DLPFC target was based on previous work,^17^ there is an absence of any validated stereotaxic target for right-sided DLPFC-rTMS; as such, we mirrored the left stereotaxic coordinate to the right hemisphere following common clinical practice. Finally, the findings at the follow-up time points must be interpreted with caution since some participants were lost to follow-up.

## Conclusions

In summary, we showed that bilateral TBS was noninferior to standard bilateral rTMS in improving depression, and similarly well tolerated, in a real-world sample of older adults with TRD. Since bilateral TBS takes 4 minutes to deliver, the use of TBS instead of standard rTMS (which takes 47.5 minutes to deliver) could increase the capacity of brain simulation programs that serve older adults. Furthermore, the short-session duration lends itself to potentially accelerated response with multiple treatments per day^42^ and the findings herein suggest that this may be possible using bilateral TBS with older adults with depression. Although there is a pervasive assumption that rTMS in general is not well suited for older adults with TRD, the results of both the THREE-D study^43^ and the present FOUR-D study indicate that rTMS outcomes in older adults can be at least as good, and perhaps better, as in younger adults. Given both its effectiveness and excellent tolerability, bilateral rTMS or TBS could be considered at an earlier stage in the treatment algorithm for older adults with TRD.

## Notes

Supplement 1. Trial protocol

Supplement 2. eTable 1. Linear Mixed-Effect Model Analysis for Depression Severity Scores from Baseline to Final Treatment and Follow-Up eTable 2. Response, Remission and Change in Depression Severity Scores from Baseline to Final Treatment with 95% CI eTable 3. Linear Mixed-Effect Model of Cognitive Outcomes from Baselines, Final and 12-Week Post Treatment

Supplement 3. Data sharing statement
