# Supplementary material for: Effectiveness of Standard Sequential Bilateral Repetitive Transcranial Magnetic Stimulation vs Bilateral Theta Burst Stimulation in Older Adults With Depression: The FOUR-D Randomized Noninferiority Clinical Trial
Source: JAMA Psychiatry. 2022 Sep 21;79(11):1065–73. doi: 10.1001/jamapsychiatry.2022.2862 (PMC9494264; doi:10.1001/jamapsychiatry.2022.2862)
Supplement: Supplement 1. — Trial protocol [file jamapsychiatry-e222862-s001.pdf]

camh Temerty Centre for Therapeutic Brain Intervention

TITLE: NEUROIMAGING PREDICTORS OF RESPONSE TO RTMS IN LATE-LIFE DEPRESSION

PRINCIPAL INVESTIGATORS:

Daniel M. Blumberger MD, MSc, FRCPC
Associate Professor, Department of Psychiatry
University of Toronto, Faculty of Medicine.
Centre for Addiction & Mental Health

Jonathan Downar MD, PhD, FRCPC
Assistant Professor, Department of Psychiatry
University of Toronto, Faculty of Medicine.
University Health Network

CO- INVESTIGATORS:

Z. Jeff Daskalakis MD, PhD, FRCPC
Benoit H. Mulsant, MD, MS, FRCPC
Tarek K. Rajji MD, FRCPC
Aristotle Voineskos MD, PhD, FRCPC
Sean Nestor MD, PhD
Moshe Isserles MD, MSc

SOURCE OF SUPPORT: CIHR

1.0 Background, Objective and Specific Aims

1.1.1 Treatment-Resistant Depression (TRD) in Older Adults

Canadians over the age of 65 constitute 13% of the population (4 million); with the aging of the baby-boomers, their number will double over the next 20 years[1]. Depression is the most common, treatable, mental disorder in late life, making it a major public health concern: 2 to 4 % of persons over the age of 65 suffer from major depression[2]. By these estimates, up to 360,000 Canadian adults over the age of 65 will suffer from major depressive disorder. These data predict an epidemic of late-life depression (LLD) that will place tremendous burden on the healthcare system. LLD is also typically complicated by co-morbid medical illness and polypharmacy and it is associated with higher rates of disability and mortality (both from suicide and physical illness)[3-5]. Older adults with depression who suffer a myocardial infarction are four times more likely to die within 4 months than those without depression[6] and the risk of a suicide attempt is double in the elderly compared to younger adults[7]. As a result, LLD is associated with higher rates of healthcare utilization and hospitalization[8-10].

Current pharmacological treatments for LLD, however, provide modest efficacy. Rates of treatment resistance in randomized controlled trials (RCT) in LLD range from 55-81% using first line antidepressants[11-14]. Further, the elderly are more likely to experience relapses and recurrences than younger adults[15-17]. The failure of first line treatment to induce remission in patients leads to impaired psychosocial function and diminished quality of life[18-20].

1.1.2 Repetitive Transcranial Magnetic Stimulation in TRD

Repetitive transcranial magnetic stimulation (rTMS) is an emerging treatment for medically refractory major depressive disorder (MDD). rTMS involves direct stimulation of cortical neurons using externally applied, powerful, focused magnetic field pulses. Dozens of studies and several meta-analyses over the last 15 years have shown that rTMS of the dorsolateral prefrontal cortex (DLPFC) produces statistically significant improvements in MDD, even when medications have failed[21-27]. In the most recent generation of randomized controlled trials, rTMS consistently achieves response rates of 50-55% and remission rates of 30-35% in medically refractory MDD patients[24,25,28-32].

rTMS has been shown to be effective and well tolerated for depression in younger and older adults[21,27,32]. However, early rTMS studies with older adults were limited by suboptimal stimulation parameters, small sample sizes and insufficient treatment durations[33-37]. A trial of rTMS in LLD with vascular disease found a greater remission rate with active compared to sham[38].

The optimal parameters for rTMS are still in the process of being established, however the most widely-used rTMS protocols apply excitatory, 10 Hz stimulation to the left DLPFC; high frequency left (HFL) or inhibitory, 1 Hz stimulation to the right DLPFC; low frequency right (LFR), or both[27,28]. Our group has studied rTMS in 43 patients with treatment resistant late-life depression (TRLLD) over two separate studies and have found superior remission rates with bilateral stimulation (40%) compared to both unilateral (0%) and sham (0%) stimulation (χ^2 = 11.30 df, 2; p = .004)[32,39]. Further evidence supporting the use of bilateral stimulation comes from another randomized control trial (RCT) conducted by our group, that assesses the efficacy of deep rTMS in

more than 60 patients (NCT01860157). The coil used in this study also has a bilateral electric field distribution. An interim analysis demonstrates superiority of the active over placebo stimulation. However, this device is not capable of "brief patterned" forms of stimulation.

1.1.3 Accelerated rTMS Protocols

Newer rTMS protocols that use "patterned" bursts of stimulation can achieve similar or more potent effects on synaptic facilitation or inhibition using markedly fewer pulses, applied over 1/10 the time of the standard form of treatment[40]. The most widely studied brief protocol is theta-burst stimulation (TBS)[41]. A blinded analysis of our multicenter non-inferiority trail (NCT01887782) demonstrates non-inferiority of intermittent TBS compared to the standard 3000 pulses FDA approved protocol in 340 younger adults with depression.

Unlike conventional stimulation, TBS mimics the endogenous theta rhythms of the brain, and this is thought to explain its greater potency in inducing long-term potentiation of synaptic connections in the target region. For example, 600 pulses of continuous TBS, applied over a period of 40 s, produces more powerful and longer-lasting inhibitory effects than 900 pulses of 1 Hz over a period of 15 minutes[40]. Likewise, 600 pulses of intermittent TBS, applied with a duty cycle of 2 s on, 8 s off, produced more powerful and longer-lasting excitatory effects than any other protocol studied in a 6-way comparison against conventional protocols[40].

Conventional rTMS protocols, as used in the major trials in the USA that secured FDA approval in 2008[21,27], applied 3000 pulses of unpatterned, 10 Hz stimulation administered over 37.5 minutes. These protocols limit the number of patients who can be treated with a single machine to around 10 per day, thus perpetuating the high cost of treatment. In contrast, the TBS protocols that have been shown effective in MDD require as little as 1-3 minutes. If shown to have non-inferior efficacy to conventional rTMS in TRLLD, these would allow for the treatment of 4-5 times as many patients per device (including turnover time), with fewer pulses of stimulation, thus reducing overall costs by a proportionate amount.

1.2.0 Predicting Treatment Response: Network Connectivity

Predicting response to treatment in TRLLD may have an even greater impact on outcomes than reducing the cost through TBS. Depression in older adults, and non-response to antidepressants, is associated with low connectivity in the cognitive control network (CCN)[41]. This network has a bilateral DLPFC distribution and is precisely the target of our stimulation protocol. Data from the resting state fMRI (rs-fMRI) of our non-inferiority trial suggests low connectivity in the left CCN at baseline predicts response to rTMS. This provides evidence that rTMS could potentially target the underlying network dysfunction in older adults and that using advanced imaging predicting tools could identify those most likely to benefit.

1.3.0 Individual-Level Prediction of Outcomes: Role of Automated Classifiers

Over two decades of neuroimaging literature are available comparing patients with MDD to healthy controls, or treatment responders to non-responders, at the group level[47-52]. The most recent studies in this

literature have also begun to identify differential predictors of outcome for two types of intervention, such as pharmacotherapy versus psychotherapy[53-55]. However, while group-level comparisons can be revealing about the general mechanisms of MDD and its various treatments, they unfortunately do not address the much more challenging problem of classifying *individual subjects* according to disease state or treatment response, in an accurate and replicable manner. Solving this problem is a crucial prerequisite for translation of predictive neuroimaging into clinical use.

Only recently has the literature begun to attempt classification of subjects at the individual level. There are a wide variety of machine learning techniques now in use to solve individual-level classification and prediction problems on high-dimensional neuroimaging datasets, including Bayesian multitask learning[56], Gaussian process classification[57], and support vector machines (SVM)[58]. The latter approach is the most widely used to date for disease state prediction.

Support vector machines (SVM) have been applied to rs-fMRI data to distinguish MDD patients with healthy controls at 95% accuracy using functional connectivity between pre-selected anatomical regions[59] and at 94.3% accuracy and 100% positive predictive value using whole-brain functional connectivity[60]. Other authors have improved classifier performance to >99% accuracy by applying graph theoretical metrics (such as modularity, participation index, or clustering coefficient) to the whole-brain functional connectivity data[61]. Similar approaches have been used to distinguish schizophrenia patients from healthy controls at 93.2% accuracy[62].

Although these figures are encouraging, it should be noted that these studies all trained their classifiers on relatively small datasets of 20-30 individuals per group, and none of them tested the performance of the models on a second replication dataset; hence, the reliability and generalizability of this approach remains unproven. One of the few studies to use a large training set (n=238) as well as replication datasets achieved 91% accuracy in classifying rs-fMRI scans as belonging to either children age 7-11 or adults age 24-30, with 92% and 93% accuracy on 2 replication datasets of sizes n=195 and n=186[63], suggesting that good replicability can be achieved if the training set is sufficiently large.

1.4.0 Objectives and Hypotheses

In summary, TBS has been shown to be effective in open-label studies of major depression. It has also been shown to have greater potency than conventional protocols in studies of motor physiology. However, to date there has been no direct comparison of the relative efficacy of these different forms of rTMS for TRLLD under randomized controlled conditions. This study will address the question of the relative efficacy of conventional versus theta-burst stimulation in the setting of TRLLD by comparing conventional HFL rTMS to TBS, while training and validating classifier algorithms for predicting treatment response using rs-fMRI.

Objective 1: To compare the effectiveness of a course of 4 minute sessions of TBS to a course of 45 minute sessions of conventional rTMS in LLD.
Hypothesis 1: Sequential bilateral forms of TBS will have non-inferior effectiveness compared to sequential bilateral conventional rTMS in older adults with depression.
Objective 2: To determine if an automated classifier can use pre-treatment resting-state fMRI, clinical and cognitive data to predict treatment outcome for both TBS and conventional rTMS.
Hypothesis 2: Patterns from whole-brain functional connectivity, obtained from pre-treatment rs-fMRI, will distinguish responders and nonresponders with >85% accuracy for TBS and standard rTMS.
Exploratory Objective 1: To compare network connectivity in depressed adults across the lifespan and rTMS-induced changes between bilateral and unilateral stimulation approaches using existing unilateral data in adults <60 from our group.

2.0 Significance

If the study hypothesis is confirmed, the implication will be that rTMS can maintain or better its current levels of efficacy using treatment protocols that can be completed in 1/10 the time of the standard form of treatment. An 8-minute treatment protocol would allow for up to 40 patients to be treated per rTMS device per day, and with a 4 week treatment protocol this would imply a capacity of nearly 500 patients per device per year. The cost of rTMS per patient could also be reduced proportionately.

In future treatment, a reliable pre-treatment fMRI test could spare non-responders from futile treatment while offering a recommendation for patients with favorable network activity patterns on fMRI. If fully implemented, this would boost the percentage of successful outcomes to 90% response and 60% remission *among treated patients*. In health-economics terms, this would reduce the cost per response. Given that responder and remitter patients would be more likely to return to work and less likely to require long-term disability support, rTMS could potentially have a positive impact on both the overall rates of treatment-resistant depression and on the significant social, economic, and health costs of the disease itself.

Our proposed trial will be large enough to demonstrate non-inferiority of TBS compared to standard rTMS, while training and validating classifier algorithms for predicting treatment response using rs-fMRI. These objectives, if realized, would yield a direct and near-term payoff in terms of outcomes and socioeconomic burden for the growing number of older adults with depression.

3.0 RESEARCH DESIGN AND METHODS

3.1.1 Research Design and Methods

Up to 220 outpatients at the University Health Network and the Centre for Addiction and Mental Health, meeting diagnostic criteria for unipolar major depressive disorder, will be randomized to receive conventional 1 Hz rTMS to the right DLPFC followed by 10Hz rTMS to the left DLPFC, or continuous theta-burst stimulation (cTBS) to the right DLPFC followed by left DLPFC intermittent theta-burst stimulation (iTBS), with rs-fMRI obtained before and after treatment. We have chosen a non-inferiority study design to align with the hypothesis of the study that TBS will be no different in terms of clinical outcome but faster and more efficient.

Following randomization, patients will undergo a series of clinical and cognitive assessments as well as an MRI (60 minute) and motor threshold testing to determine the appropriate site and strength of stimulation according to standard methods, and then begin treatment. If a participant is able to have an MRI but cannot tolerate the MRI, e.g. due to claustrophobia, they will still be allowed to undergo treatment using an approximated method called beam F3[64]. This method targets the exact same neuronavigated target and has been shown to be within 3 millimeters of the neuronavigated site. The treatment phase will last between four and six weeks. Treatment will be administered daily, 5 days per week (i.e., 20-30 treatments). Participants will be permitted to miss scheduled treatment days due to illness or scheduling conflicts. They will still receive the entire 20-30 treatments, but over a longer time period (i.e. greater than 4-6 weeks). However, those who miss more than 3 consecutive treatment days will be excluded from the study as it is our intention to encourage the commitment of the participants in the study as well as discourage prolonged duration of time taken to complete the protocol as it could affect the efficacy of the treatment. All missed treatment days will be recorded in a log for each participant.

Depressive symptoms will be assessed using the clinician-rated Montgomery Asberg Depression Rating Scale (MADRS)[65], the 17-Item Hamilton Depression Rating Scale (HRSD-17)[66], and the self-rated 16-item Quick Inventory of Depressive Symptoms ($QIDS_{16}$)[67]. Change in scores on the MADRS, from baseline to endpoint, will be the primary clinical outcome of interest. The primary neuroimaging outcome measure will be the whole-brain functional connectivity on rs-fMRI. If the subject experiences an improvement in their symptoms by a minimum of 30% based on the change in MADRS score from baseline to week 4, they will receive an additional 10 treatments over the course of 2 weeks in an attempt to optimize treatment response. Those subjects that do not achieve a 30% reduction by week 4 will be given the opportunity to continue for 2 more weeks as they may be late responders and benefit from the additional 10 treatments. All participants will undergo a post-treatment MRI scan after they complete treatment. Participants who are in remission at weeks 3 and 4 will exit the study at week 4 and undergo a post-treatment MRI scan. For participants who do not wish to continue past week 4 they will undergo a post-treatment MRI scan (30 minutes) and complete all final visit measures.

An independent, trained rater at each site will administer the clinical assessment scales at baseline, after each 5 sessions of treatment, and at 1, 4, and 12 weeks post-treatment. Cognitive assessments will be administered at baseline, after the final treatment session (including at drop-out), and at 12 weeks following the final treatment. These assessments can be done within 1-2 days of the scheduled time point. The independent raters will be blinded to the treatment being administered. All treatments will be conducted with minimization of personal contact (i.e., verbal communication) with the subject to reduce the impact of nonspecific therapeutic contact on outcomes. Patients who participate in the study will not be allowed to meet with each other before, during and after assessment or treatments in order to maintain the blind. Patients will be discontinued if they experience worsening in depression, where worsening of depression is defined as an increase in MADRS from baseline of more than 25% during two consecutive assessments, or development of active suicidal intent or attempted suicide.

Up to 90 healthy controls, age 60 and older, will undergo one MRI and cognitive assessments to serve as a comparative group for the study. All healthy controls will undergo a screening visit to assess eligibility. This will include assessment for psychiatric disorders with the MINI 6.0, current depressive symptoms as assessed by the MADRS[65], as well as current medications and a medical history using the CIRS-G[78]. If eligible, controls will

complete the same cognitive assessment battery as participants and will undergo a one hour MRI scan at Toronto Western Hospital.

Subjects will be reimbursed $150 total for the two MRI scans and as a partial contribution towards transportation, meals, time, etc. associated with their participation in the study. If subjects do not complete the second MRI they will receive $75 for their participation. Healthy controls will be reimbursed $150 for their one MRI as it is 1 hour in length and as a partial contribution towards transportation, meals, time, etc. associated with their participation in the study. They will also be reimbursed $10/hr for the cognitive assessments.

3.1.2 Subjects

<u>Inclusion Criteria:</u>

Patients will be included if they:

(1) are an outpatient
(2) are ≥60 years old
(3) have a Mini-International Neuropsychiatric Interview (MINI 6.0)[67] confirmed diagnosis of MDD, with a current MDE
(4) have failed to achieve a clinical response to an adequate dose of an antidepressant based on an Antidepressant Treatment History Form (ATHF) score of ≥ 3 in the current episode or have failed to tolerate two separate trials of an antidepressant
(5) have a score ≥ 18 on the MADRS
(6) have had no increase or initiation of any psychotropic medication in the 4 weeks prior to screening
(7) have normal electrolytes, hemoglobin and thyroid functioning based on pre-study blood work
(8) Pass the TMS adult safety screening (TASS) questionnaire
(9) Are able to have an MRI

<u>Exclusion Criteria:</u>

Patients are excluded if they:

(1) have a history of substance dependence or abuse within the last 3 months
(2) have a concomitant major unstable medical illness determined by one of the study physicians
(3) have active suicidal intent
(4) have a lifetime MINI diagnosis of bipolar I or II disorder, or primary psychotic disorder
(5) have current psychotic symptoms
(6) have a diagnosis of obsessive compulsive disorder, post-traumatic stress disorder (current or within the last year), anxiety disorder (generalized anxiety disorder, social anxiety disorder, panic disorder), or dysthymia, assessed by a study investigator to be primary. One of these comorbidities will not be exclusionary if they are not deemed to be primary.
(7) have a diagnosis of any personality disorder, and assessed by a study investigator to be primary and causing greater impairment than MDD
(8) have presumed or probable dementia or clinical evidence of dementia as assessed by a Short Blessed Test score of greater than 10.
(9) did not respond to a course of ECT in the current depressive episode

(10) have received rTMS in the current episode, patients who have had rTMS in a previous episode would be eligible
(11) have a history of a primary seizure disorder or a seizure associated with an intracranial lesion.
(12) have an intracranial implant (e.g., aneurysm clips, shunts, stimulators, cochlear implants, or electrodes) or any other metal object within or near the head, excluding the mouth, that cannot be safely removed
(13) have a implanted electronic device that is currently function such as a defibrillator
(14) currently take more than lorazepam 2 mg daily (or equivalent) or any dose of an anticonvulsant
(15) if participating in psychotherapy, must have been in stable treatment for at least 3 months prior to entry into the study, with no anticipation of change in the frequency of therapeutic sessions, or the therapeutic focus over the duration of the study
(16) non-correctable clinically significant sensory impairment (i.e., cannot hear well enough to cooperate with interview).

<u>Healthy Controls</u>
<u>Inclusion Criteria:</u>
Controls will be included if they:
(1) are ≥60 years old confirmed with a piece of identification
(2) have no psychiatric diagnoses as confirmed by the Mini-International Neuropsychiatric Interview (MINI 6.0)[67] , except for simple and specific phobias, and past substance abuse or dependence
(3) have a score ≤10 on the MADRS
(4) be on no psychotropic medication
(5) Are able to have an MRI

<u>Exclusion Criteria:</u>
Controls are excluded if they:
(1) have a history of substance dependence or abuse within the last 3 months
(2) have a concomitant major unstable medical illness determined by one of the study physicians
(3) have presumed or probable dementia or clinical evidence of dementia as assessed by a Short Blessed of greater than 10.
(4) have an intracranial implant (e.g., aneurysm clips, shunts, stimulators, cochlear implants, or electrodes) or any other metal object within or near the head, excluding the mouth, that cannot be safely removed
(5) have a implanted electronic device that is currently function such as a defibrillator
(6) non-correctable clinically significant sensory impairment (i.e., cannot hear well enough to cooperate with interview).

3.1.3 Recruitment Procedures

Potential subjects will be recruited from the outpatient geriatric psychiatry clinics at CAMH. They will also be recruited via clinician referrals. Healthy controls will be recruited via newspaper ads, and flyers placed in community clinics and throughout CAMH and surrounding areas.

3.1.4 Randomization and Blinding

Subjects will be randomized into the study, stratified by severity of treatment resistance (failure > 1 adequate trial). Degree of treatment resistance has been a major predictor of response to treatment in prior rTMS trials and it is essential to ensure that the groups are balanced with respect to this variable[21,26]. The ATHF is a validated and reliable method of assessing adequacy of prior antidepressant treatment[69,70]. We will also stratify the randomization by sites to ensure balance in the treatment condition across the two sites. We will randomize subjects based on a stratified randomization scheme using a random permuted block method. As the difference in duration between standard rTMS and TBS protocols is critical to the rationale of the study, it is not possible to blind the rTMS technicians or the patients to treatment assignment; however, since we believe there is equipoise between the two treatments and explain this to patients the expectancy effects should be minimal. **Raters** obtaining outcome measures **will be blind** to treatment assignment. All treatments will be conducted with minimization of personal contact (i.e., verbal communication) with the subject to reduce the impact of nonspecific therapeutic contact on outcomes.

3.1.5 Clinical Measures

<u>Screening and Baseline evaluation:</u>

Subjects will be screened with the MINI 6.0 to determine eligibility. The MINI assesses current and lifetime depression and other psychiatric disorders. It will be used to clarify psychiatric inclusion and exclusion criteria. The ATHF will be used to confirm eligibility based on inclusion criteria for treatment resistance. The TASS will be used to assess for potential TMS and MRI risk factors. The Short Blessed [71] will be used to screen for dementia at screening. The MoCA [72,73] will be used to further assess cognition at screening. The Beck Scale for Suicide Ideation (BSS)[73,74] will be administered to asses for suicidality. A blood test including complete blood count, liver function test and thyroid stimulating hormone will be completed prior to the screening visit in order to rule out any underlying medical causes of the depression. This process is part of the standard of care for all Temerty Centre consultations.

Healthy controls will be screened with the MINI to confirm that they do not meet criteria for current or past psychiatric disorders, except for simple/specific phobias, and past substance abuse or dependence. The TASS will be used to assess safety and eligibility to have an MRI. The Short Blessed will be used to screen for probable dementia at screening. The MADRS and HRDS-17 will be administered to assess for current depressive symptoms. Healthy controls will also complete a urine drug screen (UDS) during their screening appointment to ensure they are not using any illicit substances.

<u>Clinical Assessments During and After Treatment:</u>

Clinical outcome measures will be assessed in the week prior to treatment, after each 5 sessions of treatment, then at 1, 4, and 12 weeks after treatment. These assessments can be completed over the phone if the participant is unable to attend the visit within the given time frame. The MADRS will be the primary

outcome measure and a score of greater than or equal to 18 will used to establish eligibility. The HDRS-17 is the most commonly used outcome measure in previous randomized trials of rTMS[70,75,66] and will be included for comparison purposes. However, the MADRS is well validated in LLD and has been used in trials of other interventions in TRLLD[77] and will therefore be the primary measure. Secondary outcome measures will include the HDRS-17 and the self-rated $QIDS_{16}$. The BSS will be administered after each 5 sessions of treatment, then at 1, 4, and 12 weeks post treatment as a safety measure to assess for suicidality.

To assess other potential predictors of response of rTMS we will assess anxiety, quality of life and medical burden. We will use the Brief Symptom Inventory – Anxiety, the Quality of Life Enjoyment and Satisfaction Scale (Q-LES-Q), and the Cumulative Illness Rating Scale for Geriatrics (CIRS-G)[79].

<u>Cognitive Assessments</u>

Cognition, in particular executive function and processing speed, are often impaired in LLD[80,81]. It is an important predictor of acute antidepressant treatment outcome[82,83]. Recent data from the first multi-centre RCT in TRLLD found that set-shifting performance is an important predictor of response with augmentation pharmacotherapy[84]. rTMS may reduce the negative impact of executive dysfunction on treatment outcome through direct action on the DLPFC[85-87]. The proposed battery has been used in other LLD clinical trials and there is normative data in depressed, anxious, and healthy elderly[80-82]. Trained clinicians who are not neuropsychologists can administer the battery.

To assess executive function, we will be using 2 tests, the Delis-Kaplan Executive Function System (D-KEFS): Color-word interference [88], measuring behavioral inhibition, and the NIH Toolbox Flanker Inhibitory Control and Attention Test [89]. These 2 tests measure distinct aspects of executive functioning. They can be repeated with limited practice effects. We will use the Test of Premorbid Function to provide an estimated premorbid-IQ [90] . Memory will be assessed using the California Verbal Learning Test – second edition (CVLT-II) [90].

Neuropsychological outcomes are not a primary focus of this study, but they are important clinical predictors and may be directly modified by rTMS, thus we will carry out the battery at baseline, after four to six weeks of treatment, and at twelve weeks following the final treatment session. Healthy controls will complete the cognitive battery for comparison at the screening appointment.

3.1.6 Interventions

rTMS Treatment Procedures

Following randomization and baseline MRI, patients will undergo neuronavigation, the target location will be specified by reverse coregistration from a stereotaxic coordinate on the standard Montreal neurological Institute (MNI-152) template brain, on to each individual's anatomical MRI. The MNI coordinates for L and R DLPFC will be [x±38 y+44 z+26], drawn from a study identifying this site as optimal based on clinical outcomes and resting-state functional connectivity[90]. rTMS will employ the MagPro X100/R30 stimulator equipped with the B70 fluid-cooled coil for DLPFC stimulation (MagVenture, Farum, Denmark). Neuronavigation will proceed using the *Visor 2* system (Advanced Neuro Therapeutics, Madison, WI) to position the coil for maximal field

strength at the left and right target region of the DLPFC for each individual subject. If a participant was unable to complete the MRI they are still able to undergo treatment using beam F3[64].

Prior to the first treatment, each subject's motor threshold (MT) will first be determined according to published methods during neuronavigated TMS over the primary motor cortex[93]. Subjects will then undergo 20-30 sessions of rTMS, once daily on weekdays for 4 to 6 weeks. One study arm will undergo conventional sequential bilateral rTMS: R DLPFC stimulation at 120% resting motor threshold (RMT) at 1 Hz, in one train of 600 pulses over 10 min, followed by L DLPFC stimulation at 120% of RMT, at 10 Hz, in trains of 4 s with 26 s intertrain interval, for a total of 3000 pulses over 37.5 min. The second study arm will undergo cTBS of the R DLPFC at 120% RMT using bursts of 3 pulses at 50 Hz, bursts repeated at 5 Hz for a total of 600 pulses over 40 seconds, followed by iTBS of the L DLPFC at 120% RMT using bursts of 3 pulses at 50 Hz, bursts repeated at 5 Hz with a duty cycle of 2 s on, 8 s off for a total of 600 pulses over 3 min 9 s. Participants will be titrated to 120% RMT within the first four treatments to aid with tolerability. Subjects who cannot be titrated within 4 days will be allowed to continue in the study but the amount of time to reach the target intensity will be recorded in the treatment logs.

Common side effects of rTMS treatment are headache, discomfort and or pain at the stimulation site, facial muscle twitching, neck discomfort or pain, light-headedness or dizziness. These symptoms are expected and will be recorded separately from adverse events as side effects.

3.2 Data Collection and Statistical Considerations

3.2.1 Power calculations for primary hypotheses

Power analysis for this study draws directly upon the preliminary clinical outcome data collected in our trials using sequential bilateral rTMS in older adults. We have chosen a non-inferiority delta of 2.75 points on the MADRS as the maximum acceptable difference between groups. According to the National Institute of Clinical Health and Excellence a clinically meaningful difference between drug and placebo is greater than 3 points on a depression rating scale[94,95]. Based on data from the older adults in our two RCTs, the standard deviation of change has been 7.8 points. Setting the significance at 0.05 and the power at 80%, a sample size of 100 subjects per study arm, or **200 subjects** in total will be required to determine if cTBS then iTBS is non-inferior to 1Hz then10 Hz DLPFC-rTMS. Based on 10% attrition a total of 220 subjects will be recruited. This *n* also provides a database for classifier training that is larger than any previous study in LLD.

3.2.2 Data Collection and Management

RedCAP software will be used for data collection and overall study data management over the course of this project. RedCap is an open-source, web-based clinical data management and electronic data capture system and database. OCBN has deployed the RedCap Enterprise Edition at HPCVL, a fully supported commercial release with enhanced performance, security, validation, and quality assurance features. The system is developed and managed in compliance with HIPAA, PIPEDA, and FDA 21 CFR Part 11 regulations, providing functions such as defined user roles and privileges, user authentication and encryption, electronic signatures, de-identification of protected health information, comprehensive auditing features to record and monitor

access and changes to data, and a validated software development lifecycle. This system will be used to design electronic case report forms (eCRFs), data entry, data monitoring and cleaning, and for the query and export of datasets for statistical analysis and predictive modeling.

The forms created by the Temerty Centre for the purpose of collecting data from each subject and to track completion of assessments, MRI scans, and treatment sessions and will not contain any personal health information.

Research data gathered as part of this study may be shared and provided to other investigators affiliated with the Geriatric Mental Health Services (GMHS) for the purpose of data sharing. If participants are enrolled in multiple studies with the GMHS affiliated PIs, their research data will be shared across studies to reduce participant burden and avoid duplication of procedures. Only investigators/research teams affiliated with the PI will have access to secured files and/or research data and will be well-informed regarding the protection of participants' rights to confidentiality.

Furthermore, investigators collaborating with the study investigators (or other secondary investigators) will have access to de-identified research data collected during the study for the purposes of conducting secondary analyses about mental illnesses, such as autism spectrum disorder, depressive disorders, psychotic disorders, bipolar disorders, anxiety disorders, sleep disorders, or dementia (e.g., Alzheimer's disease). De-identified data from this study may be shared with the research community at large to advance science and health. We will remove or code any personal information that could identify participants before files are shared with other researchers to ensure that, by current scientific standards and known methods, no one will be able to identify participants from the information we share.

3.2.3 Outcome Analysis

Objective#1: Assessing non-inferiority of bilateral TBS based on change on the MADRS, an ANCOVA covarying for baseline differences will be used to measure the change at the final time point for each subject. This will be the primary clinical outcome measure.

Secondary outcome measures will include change from baseline to endpoint on the HDRS-17 and response and remission on the HDRS-17 and MADRS.

For categorical outcome measures, response will be defined as ≥ 50% reduction in symptoms on the HDRS-17. Remission will be defined as a HDRS-17 $\leq$10 and a MADRS $\leq$ 10 at the week 4 or 6 final time point. Other secondary outcome measures will explore comparison of side effects between the two treatment arms, anxiety scales, quality of life scales and cognitive measures.

Objective#2: Outcome prediction from whole-brain rs-fMRI scans, data will be analysed using the FSL platform and in-house scripts to extract whole-brain regional connectivity matrices, followed by automated classifier training, using a support vector machine approach according to previously reported methodology[60,96]. Based on preliminary data, classifiers will be trained on 4 features: whole-brain functional connectivity, region-wise betweenness centrality, and edge-wise functional connectivity variability. Automated classifiers will be trained and tested on the datasets for standard bilateral groups, with iterative cross-validation to ensure generalizability.

3.2.3 Neuroimaging Data Analysis: Preprocessing and Feature Construction

Functional neuroimaging data will be analyzed using FSL[97] for standard preprocessing steps including tissue segmentation, motion correction, co-registration of functional to anatomical scans, and spatial normalization to the MNI-152 template brain. Resting-state activity analysis will employ the aCompCor method[98] for removal of white matter and cerebrospinal fluid signal artifacts, and FSL for bandpass filtering of BOLD time-courses (0.009-0.09 Hz) followed by extraction of the signal time-course from a 9mm radius spherical region of interest (ROI) centred on the L and the R DLPFC stimulation coordinates [x±38 y+44 z+26]. This time course will be used to generate whole-brain voxelwise maps of functional connectivity to the L and R DLPFC seed ROIs in each subject.

Following our previously published methods for analyses of whole-brain connectivity as a predictor of rTMS outcome[99], we will apply a 516-region whole brain functional atlas[100] to extract time-course of activity for each region, then calculate the correlation (Pearson's *r*) of each time course to all other time-courses to create a whole-brain cross-correlation matrix for each subject, followed by application of LeDoit-Wolf shrinkage estimator and calculation of the partial correlation matrix among all regions, thus removing the effects of spurious correlations among regions due to spurious variance sources such as scanner noise or residual subject motion.

Next, this matrix will be used to construct a weighted, undirected graph of whole-brain functional connectivity. This graph will be used to calculate the normalized betweenness centrality (BC) for each brain region in each subject, using methods described in technical detail in our previously published work using this graph theoretical metric to predict rTMS treatment outcome in TRD[99].

Finally, the functional connectivity variability (FCV) will be calculated between each pair of regions in the 516-region atlas in each subject. FCV is a *dynamic* measure of functional connectivity, reflecting the stability of the correlation in activity between any two brain regions over the entire duration of the scan; a related approach is described in detail in Kucyi et al. 2013[101]. To calculate FCV for a given pair of regions, a "sliding window" of data is established using the first 15 timepoints in each data series, and the Pearson's *r* correlation calculated for this window. The window is then advanced 1 timepoint, and the process repeated, until the window reaches the end of the 300-timepoint series. The result is a series of *r*-values reflecting the *evolving* functional connectivity between the two regions over time. The variance of this *r*-series is used as the FCV. We will also explore baseline FLAIR (to assess vascular burden) and diffusion tensor imaging (to assess white matter integrity) compared to healthy controls as vascular burden has been associated with depression in late-life.[41]

In summary, the analysis methods will extract four kinds of feature maps for use in training automated classifiers for outcome prediction in each subject: i) a voxelwise map of correlation to the activity in the L and R DLPFC ROIs; ii) a whole-brain cross-correlation matrix of all 516 regions to one another; iii) BC values for each of the 516 regions; iv) FCV values for the cross-correlation of all 516 regions to one another over time.

Automated Classifier Construction

Training, testing, and cross-validation of automated classifiers will follow methods previously described for disease state classification using support vector machines (SVM). First, for each feature within the a given map, the Kendall tau rank correlation coefficient will be calculated as a non-parametric measure of the feature's relevance in discriminating responders from nonresponders. Next, features will be ranked according to discriminative power (i.e., absolute value of tau). Next, a leave-one-out cross validation approach will be used, omitting one subject per iteration during classifier training. Next, the set of consensus features (showing significant discriminative power across all 200 iterations) will be identified. This set of features will be used for construction and performance evaluation of the SVM. Classifier performance will be evaluated both in terms of overall accuracy and negative predictive value. The probability of obtaining the calculated performance under the null hypothesis will be determined using permutation testing, in which the class labels of the training data are randomly permuted and the training and cross-validation repeated, over 10 000 iterations, as previously described[60,96]. SVM performance in discriminating responders from non-responders will be assessed by this method on each of the 4 feature sets described above. The performance of each classifier will be compared using the area-under-the-curve of the receiver-operating characteristic, chi-squared tests on overall accuracy and negative predictive value[102,103], and the decision-analytical approach of Vickers[104], which accommodates a spectrum of thresholds for pursuing or foregoing treatment.

3.3 Regulatory Compliance and Monitoring

3.3.1 ClinicalTrials.gov Registration
This study will be registered on www.clinicaltrials.gov as is required by the FDA and regulatory authorities.

Table 1. Schedule of Events

Clinical and Cognitive Assessments*	Screening and Baseline	Acute Phase: Daily TBS or HFL rTMS		Follow-up Assessments	
	Week 0	Weeks 1–3 (4&5 if responder) after every 5 visits	Final Visit: Week 4 or 6 or Dropout	1 and 4 weeks following final treatment session	12 weeks following final treatment session
MINI	X				
TASS	X				
SBT	X				
MoCA	X		X		X
BSI-Anxiety	X		X		X
ATHF	X				
BSS	X	X	X	X	X
HRSD-17	X		X	X	X

MADRS	X	X	X	X	X
QIDS-16	X	X	X	X	X
Q-LES-Q	X		X		X
CIRS-G	X				
Side effect report	X	X	X		
D-KEFS Color-Word Interference	X		X		X
Test of Premorbid Function	X				
CVLT-II	X		X		X
NIH Toolbox Flanker Test	X		X		X
MRI	X		X		

ATHF: Antidepressant Treatment History Form
BSI-anxiety: Brief Symptom Inventory-anxiety subscale
BSS: Beck Suicide Scale for Suicide Ideation
CIRS-G: Cumulative Illness Rating Scale for Geriatrics
CVLT-II: California Verbal Learning Test – Second Edition
HRSD-17: 17 Item Hamilton Rating Scale for Depression
MADRS: Montgomery Asberg Depression Rating Scale
MoCA: Montreal Cognitive Assessment
MINI: Mini International Neuropsychiatric Interview
QIDS-16: 16-item Quick Inventory of Depressive Symptoms
Q-LES-Q: Quality of Life Enjoyment and Satisfaction Scale
SBT: The Short Blessed Test
TASS: Transcranial Magnetic Stimulation Adult Safety Screen.
*see proposal text for description and rationale

Table 2. Schedule of Events Healthy Controls

Clinical and Cognitive Assessments	Screening and Baseline
	Week 0
MINI	X
TASS	X
SBT	X

MoCA	X
MADRS	X
HRSD-17	X
CIRS-G	X
D-KEFS Color-Word Interference	X
Test of Premorbid Function	X
CVLT-II	X
NIH Toolbox Flanker Test	X
Urine Drug Screen	X
MRI	X

References

1. Belanger A, Martel L, Caron-Malenfant R, Statistics Canada. Demography Division. Population projections for Canada, provinces and territories, 2005-2031. Ottawa: Statistics Canada, Demography Division; 2005.
2. Blazer DG. Depression in late life: Review and commentary. Journals of Gerontology Series A-Biological Sciences and Medical Sciences 2003;58:249-65.
3. Ganguli M, Dodge HH, Mulsant BH. Rates and predictors of mortality in an aging, rural, community-based cohort: the role of depression. Arch Gen Psychiatry 2002;59:1046-52.
4. Lenze EJ, Rogers JC, Martire LM, et al. The association of late-life depression and anxiety with physical disability: a review of the literature and prospectus for future research. Am J Geriatr Psychiatry 2001;9:113-35.
5. Rovner BW, German PS, Brant LJ, Clark R, Burton L, Folstein MF. Depression and mortality in nursing homes. JAMA 1991;265:993-6.
6. Romanelli J, Fauerbach JA, Bush DE, Ziegelstein RC. The significance of depression in older patients after myocardial infarction. J Am Geriatr Soc 2002;50:817-22.
7. Minino AM, Arias E, Kochanek KD, Murphy SL, Smith BL. Deaths: final data for 2000. Natl Vital Stat Rep 2002;50:1-119.
8. Huang BY, Cornoni-Huntley J, Hays JC, Huntley RR, Galanos AN, Blazer DG. Impact of depressive symptoms on hospitalization risk in community-dwelling older persons. J Am Geriatr Soc 2000;48:1279-84.
9. Unutzer J, Tang L, Oishi S, et al. Reducing suicidal ideation in depressed older primary care patients. J Am Geriatr Soc 2006;54:1550-6.
10. Katon WJ, Lin E, Russo J, Unutzer J. Increased medical costs of a population-based sample of depressed elderly patients. Arch Gen Psychiatry 2003;60:897-903.

11. Allard P, Gram L, Timdahl K, et al. Efficacy and tolerability of venlafaxine in geriatric outpatients with major depression: a double-blind, randomized 6-month comparative trial with citalopram. *International Journal of Geriatric Psychiatry*. 2004;19(12):1123-1130.
12. Kok RM, Vink D, Heeren, TJ, et al. Lithium augmentation compared with phenelzine in treatment-resistant depression in the elderly: an open, randomized, controlled trial. *Journal of Clinical Psychiatry*. 2007;68(8):1177-1185.
13. Raskin J, Wiltse, CG, Siegal A, et al. Efficacy of duloxetine on cognition, depression, and pain in elderly patients with major depressive disorder: an 8-week, double-blind, placebo-controlled trial. *American Journal of Psychiatry*. 2007;164(6):900-909.
14. Schatzberg A and Roose S. A double-blind, placebo-controlled study of venlafaxine and fluoxetine in geriatric outpatients with major depression. *American Journal of Geriatric Psychiatry*. 2006;14(4):361-370.
15. Mulsant BH, Houck PR, Gildengers AG, et al. What is the optimal duration of a short-term antidepressant trial when treating geriatric depression? Journal of Clinical Psychopharmacology 2006;26:113-20.
16. Reynolds CF, III, Dew MA, Pollock BG, et al. Maintenance Treatment of Major Depression in Old Age. The New England Journal of Medicine 2006;354:1130-8.
17. Tew JD, Mulsant BH, Houck PR, et al. Impact of prior treatment exposure on response to antidepressant treatment in late life. American Journal of Geriatric Psychiatry 2006;14:957-65.
18. Blazer DG. The prevalence of depressive symptoms. J Gerontol A Biol Sci Med Sci 2002;57:M150-1.
19. Keller MB. Past, present, and future directions for defining optimal treatment outcome in depression: remission and beyond. JAMA 2003;289:3152-60.
20. Doraiswamy PM, Khan ZM, Donahue RMJ, Richard NE. Quality of life in geriatric depression - A comparison of remitters, partial responders, and nonresponders. American Journal of Geriatric Psychiatry 2001;9:423-8.
21. George MS, Lisanby SH, Avery D, et al. Daily left prefrontal transcranial magnetic stimulation therapy for major depressive disorder: a sham-controlled randomized trial. *Arch Gen Psychiatry*. 2010;67(5):507-516.
22. George MS, Wassermann EM, Williams WA, et al. Daily repetitive transcranial magnetic stimulation (rTMS) improves mood in depression. *Neuroreport.* 1995;6(14):1853-1856.
23. Hoflich G, Kasper S, Hufnagel A. Application of transcranial magnetic stimulation in treatment of drug-resistant major depression. *Human Psychopharmacology.* 1993;8:361-365.
24. Holtzheimer PE, 3rd, McDonald WM, Mufti M, et al. Accelerated repetitive transcranial magnetic stimulation for treatment-resistant depression. *Depress Anxiety.* Aug 23 2010.
25. Li CT, Wang SJ, Hirvonen J, et al. Antidepressant mechanism of add-on repetitive transcranial magnetic stimulation in medication-resistant depression using cerebral glucose metabolism. *J Affect Disord.* Jul 1 2010.

26. Lisanby SH, Husain MM, Rosenquist PB, et al. Daily left prefrontal repetitive transcranial magnetic stimulation in the acute treatment of major depression: clinical predictors of outcome in a multisite, randomized controlled clinical trial. *Neuropsychopharmacology.* Jan 2009;34(2):522-534.
27. O'Reardon JP et al. Efficacy and safety of transcranial magnetic stimulation in the acute treatment of major depression: a multisite randomized controlled trial. *Biol Psychiatry*. 2007;62(11):1208-1216.
28. Fitzgerald PB, Hoy K, Gunewardene R, et al. A randomized trial of unilateral and bilateral prefrontal cortex transcranial magnetic stimulation in treatment-resistant major depression. *Psychol Med.* Oct 7 2010:1-10.
29. Galletly C, Gill S, Clarke P, Burton C, Fitzgerald PB. A randomized trial comparing repetitive transcranial magnetic stimulation given 3 days/week and 5 days/week for the treatment of major depression: is efficacy related to the duration of treatment or the number of treatments? *Psychol Med.* Sep 13 2011:1-8.
30. Levkovitz Y, Harel EV, Roth Y, et al. Deep transcranial magnetic stimulation over the prefrontal cortex: evaluation of antidepressant and cognitive effects in depressive patients. *Brain Stimul.* Oct 2009;2(4):188-200.
31. McDonald WM, Durkalski V, Ball ER, et al. Improving the antidepressant efficacy of transcranial magnetic stimulation: maximizing the number of stimulations and treatment location in treatment-resistant depression. *Depression and anxiety.* Nov 2011;28(11):973-980.
32. Figiel GS, Epstein C, McDonald WM, et al. The use of rapid-rate transcranial magnetic stimulation (rTMS) in refractory depressed patients. *J Neuropsychiatry Clin Neurosci*. 1998;19(1):20-25.
33. Fregni F, Marcolin MA, Myczkowski M, et al. Predictors of antidepressant response in clinical trials of transcranial magnetic stimulation. *Int J Neuropsychopharmacol*. 2006;9(6):641-654.
34. Manes F, Jorge R, Morcuende M, et al. A controlled study of repetitive transcranial magnetic stimulation as a treatment of depression in the elderly. *Int Psychogeriatr.* 2001;13(2):225-231.
35. Mosimann UP, Marré SC, Werlen S, et al. Antidepressant effects of repetitive transcranial magnetic stimulation in the elderly: correlation between effect size and coil-cortex distance. *Arch Gen Psychiatry.* 2002;59(6):560-561.
36. Mosimann UP, Schmitt W, Greenberg BD, et al. Repetitive transcranial magnetic stimulation: a putative add-on treatment for major depression in elderly patients. *Psychiatry Res.* 2004;126(2):123-133.
37. Jorge RE, Moser DJ, Acion L, Robinson RG. Treatment of vascular depression using repetitive transcranial magnetic stimulation. *Arch Gen Psychiatry.* 2008;65(3):268-276.
38. Blumberger D et al. *J Psychiatry Neurosci.* 2016;In press.
39. Di Lazzaro V , Dileone M, Pilato F, et al. Modulation of motor cortex neuronal networks by rTMS: comparison of local and remote effects of six different protocols of stimulation. *Journal of neurophysiology.* 2011;105(5):2150-2156.

40. Huang YZ, Edwards MJ, Rounis E, et al. Theta burst stimulation of the human motor cortex. *Neuron.* 2005;45(2):201-206.
41. Alexopoulos GS, Hoptman MJ, Kanellopoulos D, et al. Functional connectivity in the cognitive control network and the default mode network in late-life depression. *Journal of affective disorders.* 2012;139(1):56-65.
42. Wilson, S. A., Lockwood, R. J., Thickbroom, G. W., & Mastaglia, F. L. (1993). The muscle silent period following transcranial magnetic cortical stimulation. *Journal of the Neurological Sciences*, *114*(2), 216–222.
43. Bajbouj, M., Lisanby, S. H., Lang, U. E., Danker-Hopfe, H., Heuser, I., & Neu, P. (2006). Evidence for impaired cortical inhibition in patients with unipolar major depression. *Biological Psychiatry*, *59*(5), 395–400. http://doi.org/10.1016/j.biopsych.2005.07.036
44. Levinson, A. J., Fitzgerald, P. B., Favalli, G., Blumberger, D. M., Daigle, M., & Daskalakis, Z. J. (2010). Evidence of cortical inhibitory deficits in major depressive disorder. *Biological Psychiatry*, *67*(5), 458–464. http://doi.org/10.1016/j.biopsych.2009.09.025
45. Bajbouj, M., Lang, U. E., Niehaus, L., Hellen, F. E., Heuser, I., & Neu, P. (2006). Effects of right unilateral electroconvulsive therapy on motor cortical excitability in depressive patients. *Journal of Psychiatric Research*, *40*(4), 322–327. http://doi.org/10.1016/j.jpsychires.2005.07.002
46. de Jesus, D. R., Favalli, G. P. de S., Hoppenbrouwers, S. S., Barr, M. S., Chen, R., Fitzgerald, P. B., & Daskalakis, Z. J. (2014). Determining optimal rTMS parameters through changes in cortical inhibition. *Clinical Neurophysiology: Official Journal of the International Federation of Clinical Neurophysiology*, *125*(4), 755–762. http://doi.org/10.1016/j.clinph.2013.09.011
47. Seminowicz DA, Mayberg HS, McIntosh AR, et al. Limbic-frontal circuitry in major depression: a path modeling metanalysis. *Neuroimage.* 2004;22(1):409-418.
48. Dougherty DD, Weiss AP, Cosgrove GR, et al. Cerebral metabolic correlates as potential predictors of response to anterior cingulotomy for treatment of major depression. *J Neurosurg.* 2003;99(6):1010-1017.
49. Mayberg HS, Brannan SK, Tekell JL, et al. Regional metabolic effects of fluoxetine in major depression: serial changes and relationship to clinical response. *Biol Psychiatry.* 2000;48(8):830-843.
50. Mayberg HS, Brannan SK, Mahurin RK, et al. Cingulate function in depression: a potential predictor of treatment response. *Neuroreport.* 1997;8(4):1057-1061.
51. Biver F, Goldman S, Delvenne V, et al. Frontal and parietal metabolic disturbances in unipolar depression. *Biol Psychiatry.* 1994;36(6):381-388.
52. Mayberg HS, Lewis PJ, Regenold W, Wagner HN, Jr. Paralimbic hypoperfusion in unipolar depression. *Journal of nuclear medicine : official publication, Society of Nuclear Medicine.* 1994;35(6):929-934.
53. McGrath CL, Kelley ME, Holtzheimer PE, et al. Toward a neuroimaging treatment selection biomarker for major depressive disorder. *JAMA Psychiatry.* 2013;70(8):821-829.
54. McGrath CL, Kelley ME, Dunlop BW, Holtzheimer Iii PE, Craighead WE, Mayberg HS. Pretreatment Brain States Identify Likely Nonresponse to Standard Treatments for Depression. *Biol Psychiatry.* 2013.

55. Kennedy SH, Konarski JZ, Segal ZV, et al. Differences in brain glucose metabolism between responders to CBT and venlafaxine in a 16-week randomized controlled trial. *Am J Psychiatry.* 2007;164(5):778-788.
56. Marquand AF, Brammer M, Williams SC, Doyle OM. Bayesian multi-task learning for decoding multi-subject neuroimaging data. *Neuroimage.* 2014.
57. Doyle OM, De Simoni S, Schwarz AJ, et al. Quantifying the attenuation of the ketamine pharmacological magnetic resonance imaging response in humans: a validation using antipsychotic and glutamatergic agents. *J Pharmacol Exp Ther.* 2013;345(1):151-160.
58. Cox DD, Savoy RL. Functional magnetic resonance imaging (fMRI) "brain reading": detecting and classifying distributed patterns of fMRI activity in human visual cortex. *Neuroimage.* 2003;19(2 Pt 1):261-270
59. Craddock RC, Holtzheimer PE, 3rd, Hu XP, Mayberg HS. Disease state prediction from resting state functional connectivity. *Magnetic resonance in medicine : official journal of the Society of Magnetic Resonance in Medicine / Society of Magnetic Resonance in Medicine.* 2009;62(6):1619-1628.
60. Zeng LL, Shen H, Liu L, et al. Identifying major depression using whole-brain functional connectivity: a multivariate pattern analysis. *Brain.* 2012;135(Pt 5):1498-1507.
61. Lord A, Horn D, Breakspear M, Walter M. Changes in community structure of resting state functional connectivity in unipolar depression. *PLoS One.* 2012;7(8):e41282.
62. Tang Y, Wang L, Cao F, Tan L. Identify schizophrenia using resting-state functional connectivity: an exploratory research and analysis. *Biomedical engineering online.* 2012;11:50
63. Dosenbach NU, Nardos B, Cohen AL, et al. Prediction of individual brain maturity using fMRI. *Science.* 2010;329(5997):1358-1361.
64. Mir-Moghtadaei A, Caballero R, Fried P, Fox MD, Lee K, Giacobbe P, Daskalakis ZJ, Blumberger DM, Downar J. *Brain Stimulation*. 2015;965-73.
65. Montgomery SA, and Asberg M. A new depression scale designed to be sensitive to change. *Br J Psychiatry*. 1979;134:382-389.
66. Hamilton M. Development of a rating scale for primary depressive illness. *The British Journal of social and clinical psychology*. 1967;6(4):278-296.
67. Rush AJ, Trivedi MH, Ibrahim HM, et al. The 16-Item Quick Inventory of Depressive Symptomatology (QIDS), clincian rating (QIDS-C), and self-report (QIDS-SR): a psychometric evaluation in patients with chronic major depression. *Biol Psychiatry*. 2003;54(5):573-583.
68. Sheehan DV, Lecrubier Y, Sheehan KH, et al. The Mini-International Neuropsychiatric Interview (M.I.N.I.): the development and validation of a structured diagnostic psychiatric interview for DSM-IV and ICD-10. *J Clin Psychiatry*. 1998;59 Suppl 20:22-33;quiz 34-57.
69. Oquendo MA, Baca-Garcia E, Kartachov A, et al. A computer algorithm for calculating the adequacy of antidepressant treatment in unipolar and bipolar depression. *J Clin Psychiatry.* 2003;64(7):825-833.
70. Sackeim HA, Prudic J, Devanand DP, et al. The impact of medication resistance and continuation pharmacotherapy on relapse following response to electroconvulsive therapy in major depression. *J Clin Psychopharmacol.* 1990;10(2):96-104.
71. Katzman R, Brown T, Fuld P, et al. Validation of a short orientation-memory concentration test of cognitive impairment. *Am J Psychiatry.*1983 140: 734-739.

72. Nasreddine ZA, Phillips NA, Bédirian V, et al. The Montreal Cognitive Assessment (MoCA): a brief screening tool for mild cognitive impairment. *J Am Geriatr Soc.* 2005;53(4):695-699.
73. Blair M, Coleman K, Jesso S, et al. "Depressive Symptoms Negatively Impact Montreal Cognitive Assessment Performance: A Memory Clinic Experience." The Canadian journal of neurological sciences. Le journal canadien des sciences neurologiques (2016): 1-5.
74. Beck AT, Kovacs M, Weissman, A. Assessment of suicidal intention: The scale of suicide ideation. *J Consult Clin Psychology.* 1979; 47: 343-352
75. Beck AT, Steer RA, Rantieri WF. Scale for suicide ideation: Psychometric properties of a self-report version. J Clin Psychology. 1988; 44:499-505
76. Petrides G, et al. ECT remission rates in psychotic versus nonpsychotic depressed patients: a report from CORE. *The Journal of ECT.* 2001;17(4):244-253.
77. Reynolds CF, 3rd, Frank E, Kupfer DJ, et al. Treatment outcomes in recurrent major depression: a post hoc comparison of elderly ("young old") and midlife patients. *Am J Psychiatry*. 1996;153(10):1288-1292.
78. Sackeim HA, Dillingham EM, Prudic J, et al. Effect of concomitant pharmacotherapy on electroconvulsive therapy outcomes: short-term efficacy and adverse effects. *Arch Gen Psychiatry*. 2009;66(7):729-737.
79. Lenze EJ, Mulsant BH, Blumberger DM, et al. Efficacy, safety, and tolerability of augmentation pharmacotherapy with aripiprazole for treatment-resistant depression in late life: a randomised, double-blind, placebo-controlled trial. *Lancet*. 2015;386(10011):2404-2412.
80. Miller MD, Paradis CF, Houck PR, et al. Rating chronic medical illness burden in geropsychiatric practice and research: application of the Cumulative Illness Rating Scale. *Psychiatry Res.* 1992;41(3):237-248.
81. Bhalla RK, Butters MA, Mulsant BH, et al. Persistence of neuropsychological deficits in the remitted state of late-life depression. *American Journal of Geriatric Psychiatry.* 2006;14(5):419-427.
82. Butters MA, Whyte EM, Nebes RD, et al. The nature and determinants of neuropsychological functioning in late-life depression. *Archives of General Psychiatry.* 2004;61(6):587-595.
83. Bogner HR, Morales KH, Post EP, et al. Diabetes, depression, and death. *Diabetes Care.* 2007;30(12):3005-3010.
84. Butters MA, Bhalla RK, Mulsant BH, et al. Executive functioning, illness course, and relapse/recurrence in continuation and maintenance treatment of late-life depression: is there a relationship?. *Am J Geriatr Psychiatry.* 2004;12(4):387-394.
85. Kaneriya SH et al. *JAMA psychiatry.* 2016;Accepted(In Press).
86. Boggio PS, Fregni F, Bermpohl F, et al. Effect of repetitive TMS and fluoxetine on cognitive function in patients with Parkinson's disease and concurrent depression. *Mov Disord.* 2005;20(9):1178-1184.
87. Martis B, Alam D, Down SM, et al. Neurocognitive effects of repetitive transcranial magnetic stimulation in severe major depression. *Clin Neurophysiol.* 2003;114(6):1125-1132.
88. O'Connor MG, Jerskey BA, Robertson EM, et al. The effects of repetitive transcranial magnetic stimulation (rTMS) on procedural memory and dysphoric mood in patients with major depressive disorder. *Cogn Behav Neurol.* 2005;18(4):223-227.
89. Delis DC, Kramer JH, Kaplan E, Holdnack J. Reliability and validity of the Delis-Kaplan Executive Function System: an update. J Int Neuropsychol Soc 2004;10:301-3.

90. Weintraub S, Dikmen SS, Heaton RK, et al. The Cognition Battery of the NIH Toolbox for Assessment of Neurological and Behavioral Function: Validation in an Adult Sample *Journal of the International Neuropsychological Society*. 2014;20(6):567–578.
91. Weschsler, D. The Tes of Premormid Functioning (TOPF). San Antonio, TX: The Psychological Corporation: 2011.
92. Woods SP, Delis DC, Scott JC, et al. The California Verbal Learning Test – second edition: Test-retest reliability, practice effects, and reliable change indices for the standard and alternate forms. *Archives of Clinical Neuropsychology.*2006; 21(5):413-420
93. Fox MD, Liu H, Pascual-Leone A. Identification of reproducible individualized targets for treatment of depression with TMS based on intrinsic connectivity. *NeuroImage*. 2012;66C:151-160.
94. Julkunen P, Säisänen L, Danner N, et al. Comparison of navigated and non-navigated transcranial magnetic stimulation for motor cortex mapping, motor threshold and motor evoked potentials. *NeuroImage*. 2009;44(3):790-795.
95. Fournier JC, DeRubeis RJ, Hollon SD, et al. Antidepressant drug effects and depression severity: a patient-level meta-analysis. *JAMA.* 2010;303(1):47-53.
96. National Collaborating Centre for Mental Health (Great Britain). *Depression in adults with a chronic physical health problem : treatment and management : National clinical practice guideline 91.* London: British Psychological Society and the Royal College of Psychiatrists; 2010.
97. Dosenbach NU, Nardo B, Cohen AL, et al. Prediction of individual brain maturity using fMRI. *Science.* 2010;329(5997):1358-1361.
98. Jenkinson M, Beckmann CF, Behrens TE, et al. FSL. *NeuroImage.* 2012;62(2):782-790.
99. Behzadi Y, Restom K, Liau J, Liu TT. A component based noise correction method (CompCor) for BOLD and perfusion based fMRI. *NeuroImage.* 2007;37(1):90-101.
100. Downar J et al. Anhedonia and reward-circuit connectivity distinguish nonresponders from responders to dorsomedical prefrontal repetitive transcranial magnetic stimulation in major depression. *Biol Psychiatry.* 2014; 76 (3): 176-185
101. Craddock RC, James GA, Holtzheimer PE, et al. A whole brain fMRI atlas generated via spatially constrained spectral clustering. *Hum Brain Mapp.* 2012;33(8):1914-1928.
102. Kucyi A et al. *Proceedings of the National Academy of Sciences of the United States of America.* 2013;110(46):18692-18697.
103. Bennett BM. On comparisons of sensitivity, specificity and predictive value of a number of diagnostic procedures. *Biometrics.* 1972;28(3):793-800.
104. Vickers AJ, Cronin AM, Gönen M. A simple decision analytic solution to the comparison of two binary diagnostic tests. *Statistics in medicine.* 2013;32(11):1865-1876.
