# Supplementary material for: Effectiveness of Standard Sequential Bilateral Repetitive Transcranial Magnetic Stimulation vs Bilateral Theta Burst Stimulation in Older Adults With Depression: The FOUR-D Randomized Noninferiority Clinical Trial
Source: JAMA Psychiatry. 2022 Sep 21;79(11):1065–73. doi: 10.1001/jamapsychiatry.2022.2862 (PMC9494264; doi:10.1001/jamapsychiatry.2022.2862)
Supplement: Supplement 2. — eTable 1. Linear Mixed-Effect Model Analysis for Depression Severity Scores from Baseline to Final Treatment and Follow-Up eTable 2. Response, Remission and Change in Depression Severity Scores from Baseline to Final Treatment with 95% CI eTable 3. Linear Mixed-Effect Model of Cognitive Outcomes from Baselines, Final and 12-Week Post Treatment [file jamapsychiatry-e222862-s002.pdf]

Supplemental Online Content

Blumberger DM, Mulsant BH, Thorpe KE, et al. Effectiveness of standard sequential bilateral repetitive transcranial magnetic stimulation vs bilateral theta burst stimulation in older adults with depression: the FOUR-D randomized noninferiority clinical trial. *JAMA Psychiatry*. Published online September 21, 2022. doi:10.1001/jamapsychiatry.2022.2862

eTable 1. Linear Mixed-Effect Model Analysis for Depression Severity Scores from Baseline to Final Treatment and Follow-Up
eTable 2. Response, Remission and Change in Depression Severity Scores from Baseline to Final Treatment with 95% CI
eTable 3. Linear Mixed-Effect Model of Cognitive Outcomes from Baselines, Final and 12-Week Post Treatment

This supplemental material has been provided by the authors to give readers additional information about their work.

eTable 1. Linear Mixed-Effect Model Analysis for Depression Severity Scores Across Time Points

Outcome Measure	n (rTMS/TBS)	rTMS (Mean ± SD)	TBS (Mean ± SD)	Estimated Adjusted Difference	Lower 90%CI#	Upper 90%CI
MADRS Baseline Score	164 (85/79)	25.6 ± 4.5	25.7 ± 4.7	----	----	----
MADRS Final Score	164 (85/79)	17.3 ± 8.9	15.8 ± 9.1	1.558	-0.86	3.97
MADRS 1-Week Post	144 (75/69)	16.1 ± 9.8	14.6 ± 9.7	1.578	-0.93	4.09
MADRS 4-Week Post	132 (69/63)	17.4 ± 10.7	13.2 ± 9.1	4.428	1.85	7.00
MADRS 12-Week Post	129 (65/64)	16.9 ± 9.4	16.0 ± 9.7	1.242	-1.35	3.83
HRSD-17 Baseline Score	164 (85/79)	18.4 ± 4.0	18.5 ± 4.7	----	----	----
HRSD-17 Final Score	164 (85/79)	12.3 ± 6.7	11.4 ± 6.8	-.924	-1.07	2.92
HRSD-17 1-Week Post	144 (75/69)	11.3 ± 6.8	10.8 ± 7.0	0.144	-1.90	2.18
HRSD-17 4-Week Post	133 (69/64)	12.4 ± 7.4	9.0 ± 6.4	3.274	1.19	5.36
HRSD-17 12-Week Post	129 (65/64)	11.9 ± 6.8	11.2 ± 6.5	0.807	-1.30	2.91
QIDS-16 Baseline Score	164 (85/79)	15.7 ± 4.4	15.9 ± 4.4	----	----	----
QIDS-16 Final Score	164 (85/79)	10.8 ± 6.0	9.7 ± 6.1	1.315	-0.20	2.83
QIDS-16 1-Week Post	144 (75/69)	9.5 ± 6.3	9.4 ± 6.3	0.128	-1.44	1.70
QIDS-16 4-Week Post	133 (70/63)	10.5 ± 6.3	8.1 ± 5.1	1.953	0.34	3.56
QIDS-16 12-Week Post	126 (64/62)	10.7 ± 6.1	9.7 ± 5.0	0.416	-1.22	2.05
BSI-A Baseline	164 (85/79)	11.1 ± 5.6	9.8 ± 5.2	----	----	----
BSI-Final	164 (85/79)	6.6 ± 5.8	5.7 ± 5.2	0.262	-1.28	1.81
BSI 12-Week Post	129 (65/64)	6.8 ± 5.8	5.2 ± 3.8	0.855	-0.70	2.47

Continuous variables are denoted as average ± standard deviation. Results include baseline adjusted estimates. *rTMS, standard bilateral protocol. TBS, bilateral theta-burst stimulation. MADRS, Montgomery-Asberg Depression Rating Scale; HRSD-17, 17-item Hamilton Rating Scale for Depression; QIDS-16, 16-item Quick Inventory of Depressive Symptomatology, Self-Rated; BSI-A, Brief Symptom Inventory – Anxiety Subscale.* # lower 95% CI of one-sided test for non-inferiority.

eTable 2. Response, Remission and Change in Depression Severity Scores from Baseline to Final Treatment with 95% CI

Outcome Measure	rTMS (n=85) (Mean ± SD) % (n)	TBS (n=79) (Mean ± SD) % (n)	Estimated Adjusted Difference	Lower 95%CI[#]	Upper 95%CI	Significance[#]
MADRS Baseline Score	25.6 ± 4.5	25.7 ± 4.7	----	----	----	----
MADRS Final Score	17.3 ± 8.9	15.8 ± 9.1	1.547	-1.08	4.18	0.0008
MADRS Response Rate	32.9 (28)	44.3 (35)	11.4%	-3.5%	26.2%	0
MADRS Remission Rate	32.9 (28)	35.4 (28)	2.5%	-12.0%	17.0%	0.046
HRSD-17 Baseline Score	18.4 ± 4.1	18.5 ± 4.7	----	----	----	----
HRSD-17 Final Score	12.3 ± 6.8	11.4 ± 6.8	0.917	-1.04	2.87	0.0002
HRSD-17 Response Rate	29.6 (24)	41.9 (31)	12.3%	-2.7%	27.3%	0
HRSD-17 Remission Rate	27.2 (22)	33.8 (25)	6.6%	-7.9%	21.1%	0.012
QIDS-16 Baseline Score	15.7 ± 4.4	15.9 ± 4.4	----	----	----	----
QIDS-16 Final Score	10.8 ± 6.0	9.7 ± 6.1	1.268	-0.37	2.94	1.6×10^{-6}
QIDS-16 Response Rate	35.7 (30)	44.3 (35)	8.6%	-6.4%	23.6%	0.001
QIDS-16 Remission Rate	21.4 (18)	31.6 (25)	10.2%	-3.3%	23.7%	0.002
BSI-A Baseline Score	11.1 ± 5.6	9.8 ± 5.2	----	----	----	----
BSI-Final Score	6.6 ± 5.8	5.7 ± 5.2	0.341	-1.24	1.93	8.8×10^{-5}

Continuous variables are denoted as average ± standard deviation. For estimated adjusted difference values, positive values indicate greater change in the TBS group, while negative values indicate greater change in the bilateral rTMS group. *p*-values indicate the significance of rejecting the null hypothesis based on the change in symptoms in the two groups and on a non-inferiority δ of 15% for the proportion of responders and 10% for the proportion of remitters. *rTMS, standard bilateral protocol. TBS, bilateral theta-burst stimulation. MADRS, Montgomery-Asberg Depression Rating Scale HRSD-17, 17-item Hamilton Rating Scale for Depression; QIDS-16, 16-item Quick Inventory of Depressive Symptomatology, Self-Rated; BSI-A, Brief Symptom Inventory – Anxiety Subscale. #* corresponds to the lower 97.5% CI of the one-sided test for non-inferiority.

eTable 3. Linear Mixed-Effect Model of Cognitive Outcomes Across Time Points

Outcome Measure	ITT n (rTMS/TBS)	rTMS (Mean ± SD)	TBS (Mean ± SD)	Estimated Adjusted Difference	Lower 90%CI	Upper 90%CI
Flanker Baseline	172 (87/85)	89.1 ± 10.3	89.5 ± 9.0	----	----	----
Flanker Final	148 (79/69)	90.8 ± 10.0	90.6 ± 9.6	0.401	-1.61	2.41
Flanker 12-Week Post	107 (58/49)	93.4 ± 7.7	92.7 ± 9.1	0.443	-1.82	2.71
DKEFS Colour Naming Baseline	172 (87/85)	8.6 ± 3.6	9.5 ± 3.0	----	----	----
DKEFS Colour Naming Final	150 (81/69)	9.0 ± 3.5	9.6 ± 3.1	0.152	-0.49	0.79
DKEFS Colour Naming 12-Week Post	106 (58/48)	9.6 ± 3.1	10.1 ± 3.1	0.268	-0.47	1.01
DKEFS Colour Word Baseline	172 (87/85)	10.3 ± 3.0	10.6 ± 2.4	----	----	----
DKEFS Colour Word Final	150 (81/69)	10.1 ± 3.0	10.4 ± 2.8	0.097	-0.50	0.69
DKEFS Colour Word 12-Week Post	106 (58/48)	10.3 ± 2.9	10.5 ± 2.4	0.332	-0.33	0.99
DKEFS Inhibition Baseline	169 (85/84)	10.1 ± 2.9	10.4 ± 2.9	----	----	----
DKEFS Inhibition Final	150 (81/69)	10.4 ± 2.8	11.0 ± 2.9	-0.106	-0.63	0.41
DKEFS Inhibition 12-Week Post	104 (57/47)	11.4 ± 2.5	11.6 ± 2.5	0.202	-0.40	0.80
DKEFS Inhibition/Switching Baseline	169 (85/84)	10.7 ± 2.9	11.1 ± 2.5	----	----	----
DKEFS Inhibition/Switching Final	147 (78/69)	11.3 ± 2.7	11.5 ± 2.7	0.046	-0.49	0.58
DKEFS Inhibition/Switching 12-Week Post	104 (57/47)	11.8 ± 2.6	11.8 ± 2.5	0.503	-0.10	1.11
CVLT-II Trials 1–5 Baseline	168 (84/84)	47.2 ± 10.7	47.8 ± 10.9	----	----	----
CVLT-II Trials 1–5 Final	150 (79/71)	57.1 ± 13.3	57.5 ± 12.8	1.217	-1.86	4.29
CVLT-II Trials 1–5 12-Week Post	111 (61/50)	57.9 ±13.7	60.4 ± 12.7	-0.102	-3.53	3.33
CVLT-II Short Delay Free Recall Baseline	168 (84/84)	-0.2 ± 1.1	-0.1 ± 1.2	----	----	----
CVLT-II Short Delay Free Recall Final	150 (79/71)	0.4 ± 1.2	0.5 ± 1.3	0.077	-0.20	0.36
CVLT-II Short Delay Free Recall 12-Week Post	111 (61/50)	0.5 ± 1.3	0.7 ± 1.2	0.021	-0.29	0.33
CVLT-II Long Delay Free Recall Baseline	168 (84/84)	-0.3 ± 1.1	-0.2 ± 1.2	----	----	----
CVLT-II Long Delay Free Recall Final	149 (78/71)	0.3 ± 1.2	0.3 ± 1.2	0.096	-0.17	0.37
CVLT-II Long Delay Free Recall 12-Week Post	111 (61/50)	0.3 ± 1.4	0.6 ± 1.3	-0.037	-0.34	0.27
MOCA Baseline	171 (86/85)	25.0 ± 3.2	25.1 ± 3.3	----	----	----
MOCA Final	156 (83/73)	24.9 ± 3.5	25.6 ± 2.9	-0.58	-1.35	0.20
MOCA 12-Week Post	120 (64/56)	24.7 ± 2.7	24.8 ± 3.3	-0.20	-1.06	0.67

Continuous variables are denoted as average ± standard deviation. Results include baseline adjusted estimates. *rTMS, standard bilateral protocol. TBS, bilateral theta-burst stimulation. ITT- Intent-to-treat. DKEFS, Delis–Kaplan Executive Function System; CVLT, California Verbal Learning Test; MOCA, Montreal Cognitive Assessment.*
