# Supplementary material for: Effectiveness of Standard Sequential Bilateral Repetitive Transcranial Magnetic Stimulation vs Bilateral Theta Burst Stimulation in Older Adults With Depression: The FOUR-D Randomized Noninferiority Clinical Trial
Source: JAMA Psychiatry. 2022 Sep 21;79(11):1065–73. doi: 10.1001/jamapsychiatry.2022.2862 (PMC9494264; doi:10.1001/jamapsychiatry.2022.2862)
Supplement: Supplement 3. — Data sharing statement [file jamapsychiatry-e222862-s003.pdf]

Data Sharing Statement

Blumberger. Effectiveness of Standard Sequential Bilateral Repetitive Transcranial Magnetic Stimulation vs Bilateral Theta Burst Stimulation in Older Adults With Depression. *JAMA Psychiatry*. Published September 21, 2022. doi:10.1001/jamapsychiatry.2022.2862

Data
Data available: Yes
Data types: Deidentified participant data
How to access data: daniel.blumberger@camh.ca
When available: beginning date: 01-01-2024, end date: 12-31-2027

Supporting Documents
Document types: None

Additional Information
Who can access the data: Researchers whose proposed use of the data has been approved.
Types of analyses: Researchers who provide a methodologically sound proposal that includes a protocol and a statistical analysis plan, and is not in conflict with the investigators' publication plan.
Mechanisms of data availability: To gain access, data requestors will need to sign a data access agreement.
